# Upregulation of peroxisome proliferator-activated receptor-α and the lipid metabolism pathway promotes carcinogenesis of ampullary cancer

**DOI:** 10.7150/ijms.48123

**Published:** 2021-01-01

**Authors:** Chih-Yang Wang, Ying-Jui Chao, Yi-Ling Chen, Tzu-Wen Wang, Nam Nhut Phan, Hui-Ping Hsu, Yan-Shen Shan, Ming-Derg Lai

**Affiliations:** 1 PhD Program for Cancer Molecular Biology and Drug Discovery, College of Medical Science and Technology, Taipei Medical University, Taipei 11031, Taiwan.; 2 Graduate Institute of Cancer Biology and Drug Discovery, College of Medical Science and Technology, Taipei Medical University, Taipei 11031, Taiwan.; 3 Department of Surgery, National Cheng Kung University Hospital, College of Medicine, National Cheng Kung University, Tainan 70403, Taiwan.; 4 Institute of Clinical Medicine, College of Medicine, National Cheng Kung University, Tainan 70101, Taiwan.; 5 Senior Citizen Service Management, Chia-Nan University of Pharmacy and Science, Tainan 71710, Taiwan.; 6 NTT Institute of Hi-Technology, Nguyen Tat Thanh University, Ho Chi Minh City, Vietnam.; 7 Department of Biostatistics, Vanderbilt University Medical Center, Nashville, TN 37232, USA.; 8 Department of Biochemistry and Molecular Biology, College of Medicine, National Cheng Kung University, Tainan 70101, Taiwan.; 9 Institute of Basic Medical Sciences, National Cheng Kung University, Tainan 70101, Taiwan.; 10 Center for Infectious Diseases and Signaling Research, College of Medicine, National Cheng Kung University, Tainan 70101, Taiwan.

**Keywords:** Ampullary cancer, Lipid metabolism, Carcinogenesis, *PPARA* gene, Bioinformatics

## Abstract

Ampullary cancer is a rare periampullary cancer currently with no targeted therapeutic agent. It is important to develop a deeper understanding of the carcinogenesis of ampullary cancer. We attempted to explore the characteristics of ampullary cancer in our dataset and a public database, followed by a search for potential drugs. We used a bioinformatics pipeline to analyze complementary (c)DNA microarray data of ampullary cancer and surrounding normal duodenal tissues from five patients. A public database from the National Center for Biotechnology Information Gene Expression Omnibus (NCBI GEO) was applied for external validation. Bioinformatics tools used included the Gene Set Enrichment Analysis (GSEA), Database for Annotation, Visualization and Integrated Discovery (DAVID), MetaCore, Kyoto Encyclopedia of Genes and Genomes (KEGG), Hallmark, BioCarta, Reactome, and Connectivity Map (CMap). In total, 9097 genes were upregulated in the five ampullary cancer samples compared to normal duodenal tissues. From the MetaCore analysis, genes of peroxisome proliferator-activated receptor alpha (*PPARA*) and retinoid X receptor (*RXR*)-regulated lipid metabolism were overexpressed in ampullary cancer tissues. Further a GSEA of the KEGG, Hallmark, Reactome, and Gene Ontology databases revealed that *PPARA* and lipid metabolism-related genes were enriched in our specimens of ampullary cancer and in the NCBI GSE39409 database. Expressions of *PPARA* messenger (m)RNA and the PPAR-α protein were higher in clinical samples and cell lines of ampullary cancer. US Food and Drug Administration (FDA)-approved drugs, including alvespimycin, trichostatin A (a histone deacetylase inhibitor), and cytochalasin B, may have novel therapeutic effects in ampullary cancer patients as predicted by the CMap analysis. Trichostatin A was the most potent agent for ampullary cancer with a half maximal inhibitory concentration of < 0.3 μM. According to our results, upregulation of *PPARA* and lipid metabolism-related genes are potential pathways in the carcinogenesis and development of ampullary cancer. Results from the CMap analysis suggested potential drugs for patients with ampullary cancer.

## Introduction

Ampullary cancer is a rare type of cancer with an incidence of 0.0004%~0.0006% in the general population and 0.5% among total gastrointestinal cancer cases 1. The 5-year survival rate of ampullary cancer patients ranges 30%~50%, and adjuvant chemotherapy or radiotherapy fails to improve survival 2-4. Adjuvant therapy provides only small benefits for ampullary cancer patients with poor prognostic factors 5. Certain regimens of chemotherapy may be beneficial in advanced or metastatic ampullary cancers 6. Therefore, new therapeutic agents for ampullary cancer patients imperatively need to be discovered.

In recent years, mechanisms of carcinogenesis have been extensively studied in cancer research. Several potential pathways were examined in ampullary cancer 7,8. Previous research suggested a correlation between survival of ampullary cancer patients and expressions of membrane receptors for bile acids 9. Genetic polymorphisms of lipid metabolism-related genes were correlated with the development of biliary tract cancer and ampullary cancer 10,11. The mechanism of development of ampullary cancer remains inexplicit, and comprehensive analyses are required.

In cancer research, databases are used to speculate on disease-drug-gene interactions and discover potential therapeutic agents 12. The Connectivity Map (CMap) comprises over 1300 drugs and small molecules approved by the US Food and Drug Administration (FDA). The CMap provides information about gene expression patterns of four types of cancer cell lines treated with FDA-approved drugs and/or small molecules 13. In the present study, we exploited an Agilent microarray to compare gene expressions in ampullary cancer tissues to those of surrounding normal duodenal tissues. MetaCore was utilized to analyze potential signaling pathways in ampullary cancer patients. Based on our bioinformatics analysis, we found that peroxisome proliferator-activated receptor (PPAR) and its related lipid metabolism pathway were involved in the development of ampullary cancer. The PPAR family protein, known as ligand-inducible transcription factors, comprises three members: PPAR-α, PPAR-β, and PPAR-γ (*PPARA*, *PPARD*, and *PPARG* gene, respectively). These are members of the nuclear hormone receptor superfamily and can be partially distinguished by their tissue distributions, ligands, and target specificities 14-16. Depending on the type of ligand or tissue of origin, activation of PPAR-α either strengthens or attenuates tumor progression 17. Although PPAR-α acts as a key factor of tumorigenesis, its role in ampullary cancer is unknown. The aim of the present study was to explore the function of PPAR-α in ampullary cancer from our dataset and public databases, followed by a search for potential drugs using CMap. These results were confirmed in cell lines and clinical samples of ampullary cancer.

## Materials and methods

### Study design and patient population

Five patients with an ampullary adenocarcinoma (two women with T2N0, stage IB cancer and three men with T3N0, stage IIA cancer) who underwent a radical resection in our hospital from 2003 to 2012 were selected. The pathological stage followed guidelines defined by the American Joint Committee on Cancer (AJCC) *Cancer Staging Manual*, 7^th^ edition 18. Fresh specimens of ampullary cancer and surrounding normal duodenal tissues were collected. General characteristics of patients such as demographics, histopathological findings, and outcomes were collected by a retrospective chart review (Supplementary Table 1). Patients received regular follow-up and routine examinations every 3~6 months according to the clinician's suggestion. Formal written informed consent was obtained. This study was approved by the Institutional Review Board of our hospital (NCKUH IRB no. A-ER-100-395).

### Total RNA extraction and microarray analysis of gene expressions

TRIzol reagent (Invitrogen, Carlsbad, CA, USA) was used to extract total RNA. The RNA integrity of each sample was evaluated by an Agilent 2100 Bioanalyzer (Agilent, Santa Clara, CA, USA). A MaestroNano spectrophotometer (Maestrogen, Las Vegas, NV, USA) was used to check if A260/A230 and A260/A280 ratios of RNA ranged 1.8~2.1. Fresh samples of ampullary cancer and surrounding normal duodenal tissues were analyzed by a complementary (c)DNA microarray. During the *in vitro* transcription process, RNA from normal samples was labeled with Cy3, and that from ampullary adenocarcinomas was tagged with Cy5 dye (PerkinElmer, Waltham, MA, USA). An Agilent SurePrint G3 Human GE 8×60K microarray was hybridized with Cy-labeled cDNA. The scanning wavelength of the microarray was set to 535 nm for Cy3 and 625 nm for Cy5. Lowes' method with a rank-consistency filter was used to normalize scanned images. Data were analyzed with Agilent Genespring software as we previously described 19-22.

### Bioinformatics analyses

MetaCore was used to construct biological networks and associated diseases from expressed gene sets in the cDNA microarray. The cutoff level was set to a *p* value of < 0.05 to determine significant enrichment in the MetaCore database. Gene Set Enrichment Analysis (GSEA) software was applied for enrichment of gene sets that had a common biological function, chromosomal location, or regulation 23,24. The Database for Annotation, Visualization and Integrated Discovery (DAVID) was used to perform enrichment analyses for signal pathways 25-27. We used Fisher's test to select significantly enriched pathways in ampullary cancer. Statistical significance of Gene Ontology (GO) terms was set to a *p* value of < 0.05. REVIGO software was used to remove any redundancy of GO terms 28,29. Simulation of Multivariate Linear Model Data (SimRel) for measuring semantic similarity was used with a median of 0.7 for similarity between enriched GO terms. A list of potential genes was mapped to the Kyoto Encyclopedia of Genes and Genomes (KEGG), Hallmark, BioCarta, and Reactome for the biological pathway analysis, with a* p* value of < 0.05 accepted as being statistically significant 30-32. A flowchart of the analysis is summarized in Supplementary Figure 1.

### Drug response profiles

The CMap, a huge database containing 7000 expression profiles of human cell lines treated with more than 1300 compounds, was exploited to speculate on drug responses after overexpression of particular genes. Five hundred of the most and least highly expressed genes from the cDNA microarray of ampullary cancer were input to CMap to predict potential drugs. To reveal meaningful biological connections of the gene list, the gene-signature perturbation approach was performed in the present study 33,34. A two-sided *t*-test was applied to classify the effectiveness of compounds for differential gene expressions. Chemicals with a negative correlation with ampullary cancer were classified by a standardized connection score, perturbation stability, and *p* value.

### Cell lines

The TGBC-18 TKB cell line was established from human cancer of the papilla of Vater and was obtained from the RIKEN Bioresource Center (Ibaraki, Japan). Importation of a cancer cell line was complied with national legislation and was approved by the Centers for Disease Control, Taiwan. The SNU-478 cell line derived from an ampullary adenocarcinoma was a kind gift from Prof. Li-Tzong Chen (National Institute of Cancer Research, Taiwan). Pancreatic cancer cell lines (Pan1 and MIA-Pan2), gastric cancer cell lines (AGS and MKN45), and a colon cancer cell line (HCT-116) were obtained from the Bioresource Collection and Research Center at the Food Industry Research and Development Institute (Hsinchu, Taiwan). Cells were maintained in Dulbecco's modified Eagle's medium with 10% heat-inactivated fetal bovine serum.

### Real-time quantitative polymerase chain reaction (qPCR) and reverse-transcription (RT)-PCR

Total RNA was extracted from cultured cells using a RNeasy Mini kit (Qiagen, Hilden, Germany) and single-stranded cDNA was synthesized from total RNA using M-MuLV reverse transcriptase (Roche, Basel, Switzerland) and oligo-dT random primers. cDNA was amplified with primers for specific genes, and an RT-PCR was performed with 35 cycles of denaturing for 30 s at 94 ºC, annealing for 45 s at 60 ºC, and extension for 30 s at 72 ºC in a commercial PCR system. The RT-PCR primers are listed below: *PPARA* forward, 5'-TCT GGC CAA GAG AAT CTA CGA G-3' and reverse, 5'-CAG CCA TAC ACA GTG TCT CCA T-3' and actin forward, 5'-AGC GGG AAA TCG TGC GTG-3' and reverse, 5'-CAG GGT ACA TGG TGG TGG TGC C-3'. PCR products were visualized after electrophoresis on a 1.9% agarose gel and stained with SAFE DNA gel stain (Invitrogen). Bands on the gels were quantified by a densitometric analysis, normalized relative to the β-actin band, and compared to the control group. For the qPCR, specific primers and cyber-green probes were selected. qPCR parameters were as follows: 45 cycles of 95 ºC for 10 min, 95 ºC for 10 s, 60 ºC for 30 s, and 72 ºC for 1 s, followed by 40 ºC for 30 s. The increase in the fluorescence of fluorescein was automatically measured during the PCR. All samples were amplified in duplicate, and the C_T_ value was recorded. The 2-Δ-Δ C_T_ value was calculated following *GAPDH* normalization. The qPCR primers were as follows: *ACAA1* forward, 5'-TGA CAG TGA GTG ACG TGG AC-3' and reverse, 5'-TTC CAG TCC CGA TGC ACA TG-3'; *FABP1* forward, 5'-TGC CAC CAT GAG TTT CTC CG-3' and reverse, 5'-GAT TTC CGA CAC CCC CTT G-3'; *FABP2* forward, 5'-AAC GGA CAG ACA ATG GAA AC-3' and reverse, 5'-CGC CAA GAA TAA TGC TCA ATC-3'; *PPARA* forward, 5'-GGC GAG GAT AGT TCT GGA AG-3' and reverse, 5'-AGG ATA AGT CAC CGA GGA GG-3; and *GAPDH* forward, 5'-AGC CAC ATC GCT CAG ACA C-3' and reverse, 5'-GCC CAA TAC GAC CAA ATC C-3'.

### Cell proliferation assay

Cancer cells were seeded into each well of 96-well plates a day prior to treatment. After treatment with alvespimycin, cytochalasin B, or trichostatin A, 180 μL of a 3-(4,5-dimethylthiazol-2-yl)-2,5-diphenyltetrazolium bromide (MTT) solution was added to each well and incubated for 4 h. The absorbance of the solution was quantified by measuring at a 595-nm wavelength with a spectrophotometer.

### Immunohistochemical (IHC) staining

Formalin-fixed paraffin-embedded sections of ampullary cancer were obtained from the Human Biobank of National Cheng Kung University Hospital. Slides were deparaffinized, rehydrated, and underwent heat-retrieval in a pressure boiler. Expression of the PPAR-α protein was detected by a polyclonal rabbit anti-PPAR-α antibody (GeneTex, Irvine, CA, USA) and a goat anti-rabbit secondary antibody conjugated with a peroxidase-labeled polymer (EnVisionTM System, Dako, Denmark). Color was developed with 3-amino-9-ethyl carbazole (Zymed, Waltham, MA, USA), and nuclei were counterstained with Mayer's hematoxylin. Slides were evaluated as low or high expression of PPAR-α.

### Statistical analysis

All statistical analyzes were performed with STATA 16 (StataCorp, College Station, TX, USA). The nonparametric Kruskal-Wallis test was utilized for continuous variables. The half maximal inhibitory concentration (IC_50_) was calculated and graphed using PRISM 5.0 (GraphPad, San Diego, CA, USA). The level of statistical significance was set to *p* < 0.05.

## Results

### PPAR and the lipid metabolism-related pathway in ampullary cancer

**Figure 1:**
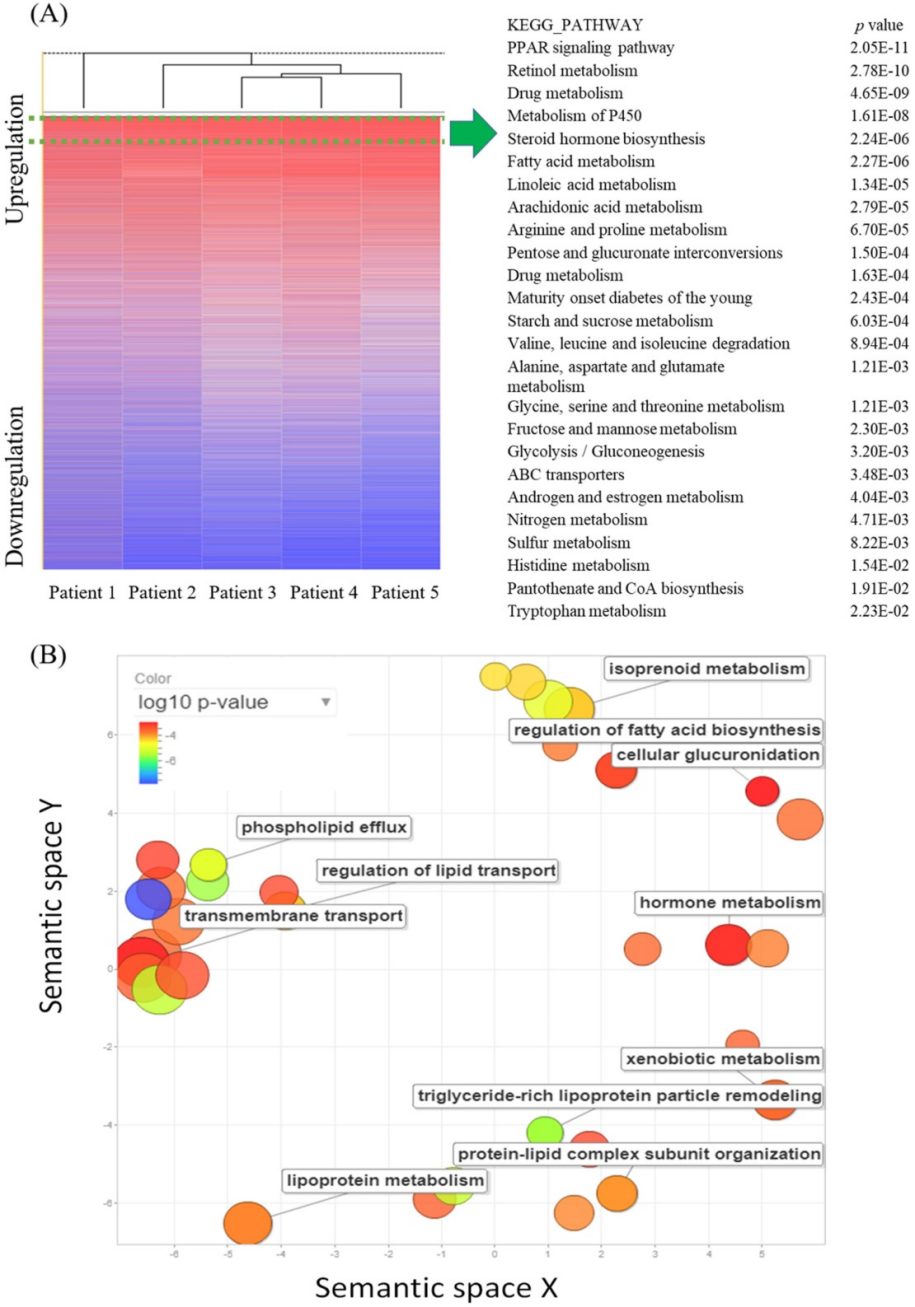
** Complementary (c)DNA microarray comparing gene expressions in ampullary cancer with those of normal duodenal tissues.** (A) The heatmap shows gene rankings from the highest to lowest after normalization. The peroxisome proliferator-activated receptor (PPAR) signaling pathway had the highest expression. The top 25 Kyoto Encyclopedia of Genes and Genomes (KEGG) pathways are listed with their *p* values. (B) Gene Ontology (GO) scatterplot constructed by REVIGO. Individual circles indicate representative clusters. The color of the circles indicates *p* values of the GO analysis (legend in the upper left-hand corner). The size of the circles indicates the frequency of the GO term in the underlying database; the larger the circle, the higher the frequency of gene expression.

The cDNA microarray consisting of five pairs of ampullary cancer and normal duodenal tissues was screened to identify novel pathways with an absolute multiple of change of cancer-to-normal ratio of > 1.5. In total, 9097 genes were upregulated in the five cancer specimens, while 4891 genes were downregulated. In a comparison of the recurrence status of ampullary cancer patients, we found that 675 genes were upregulated in patients with recurrence, whereas 895 genes had high expression in patients without recurrence. The 9097 upregulated genes in all five specimens were uploaded to the online DAVID software and compared with KEGG pathways to match gene signatures. Table 1 and Supplementary Table 2 list enriched pathways of upregulated genes in ampullary cancer as analyzed by the KEGG. Upregulated genes were enriched in the PPAR signaling pathway, retinol metabolism, drug metabolism, metabolism of P450, and steroid hormone biosynthesis (Figure 1A). We used REVIGO to summarize long lists of GO terms from overexpressed genes in the cDNA microarray. Based on semantic similarities after merging and replacing the representative subset, non-redundant GO terms were displayed in a semantic space with a cutoff point of 1% (Figure 1B). Relevant biological processes comprised regulation of fatty acid biosynthesis, metabolism of hormones or lipoproteins, the cellular transport system, and lipid catabolic processes. Detailed GO terms and related genes are listed in Supplementary Table 3.

### MetaCore pathway analysis of gene expression in ampullary cancer

**Figure 2:**
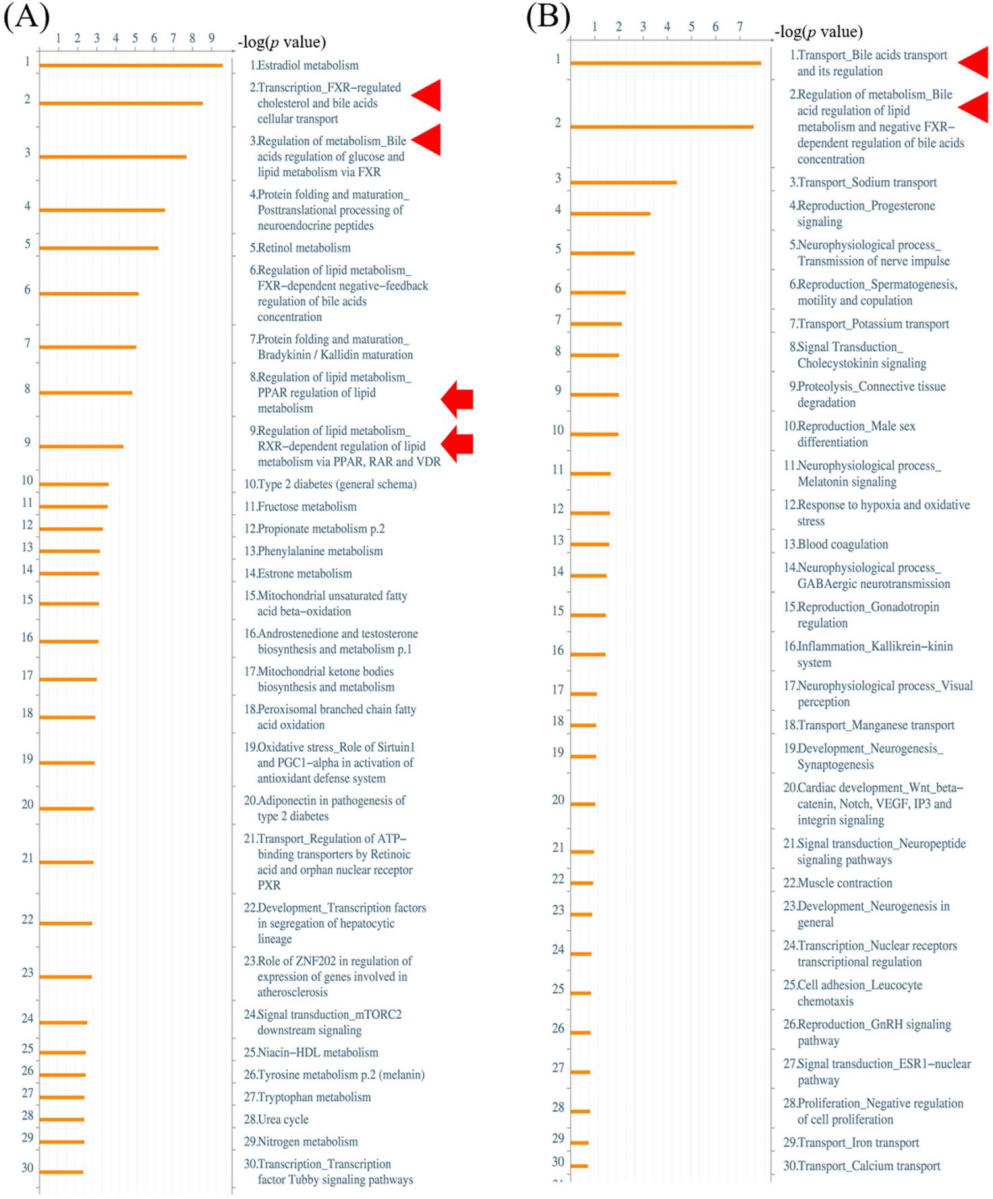
** MetaCore analysis of a complementary (c)DNA microarray of ampullary cancer.** (A) Multiples of altered gene expressions between ampullary cancer and normal duodenal tissues are classified according to GeneGO PATHWAY maps. The list begins with those with the lowest *p* values, and the top 5% are shown. (B) GeneGO NETWORKS analysis indicated that differentially expressed genes were highly correlated with networks of signal pathways. The 30 most significant networks are listed. Bile acid-associated pathways are marked with arrowheads and lipid metabolism-related signal pathways with arrows.

**Figure 3:**
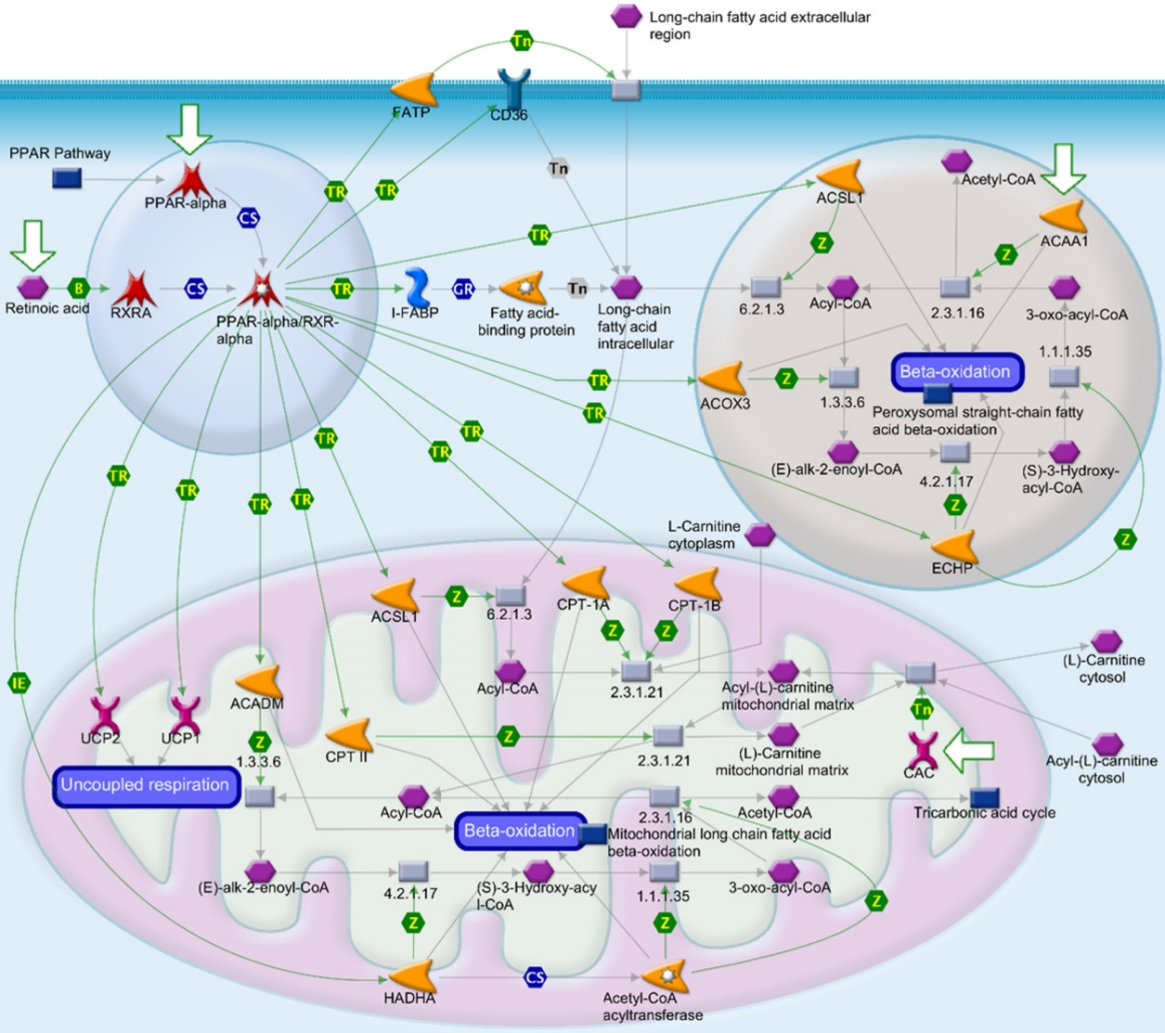
** Peroxisome proliferator-activated receptor (PPAR) signaling pathway in the MetaCore analysis.** The network begins from PPAR (upper left-hand corner) and expands to the entire cell. B, binding; CM, covalent modification; +P, phosphorylation; T, transformation; Tn, transport; Z, catalysis; TR, transcription regulation; IE, influence on expression; GR, group relation; CS, complex subunit. A green arrow represents (positive) activation of the process. A red arrow represents (negative) inhibition of the process. A gray arrow represents an unspecified process.

Alterations of gene expressions between ampullary cancer and normal duodenal tissues were analyzed using the MetaCore/GeneGO database. MetaCore contains various biomolecular interactions, such as protein-protein, protein-DNA, and protein-metabolite interactions, with relevant signal pathways. The top 5% of significantly differentially expressed genes (DEGs) between paired samples of ampullary cancer and matched normal tissues were uploaded to MetaCore. Based on upregulated genes in ampullary cancer, transport and regulation of bile acid was the most significant pathway with a *p* value of 2.757 × 10^-9^ (arrowhead in Figure 2A, B). This result was consistent with previous studies suggesting the importance of the bile acid-related pathway in ampullary cancer 9. Moreover, MetaCore results indicated that “Regulation of lipid metabolism PPAR regulation of lipid metabolism (*p* = 1.592 × 10^-5^) and “Regulation of lipid metabolism RXR-dependent regulation of lipid metabolism via PPAR, RAR and VDR” (RAR, retinoic acid receptor; VDR, vitamin D receptor; *p* = 4.734 × 10^-5^) ranked in the top significant pathways in ampullary cancer (arrow in Figure 2A). The PPAR-α/RXR-α signaling pathways act in energy provision, mitochondrial metabolism, and cellular homeostasis. Multiples of change of gene expressions between ampullary cancer and normal duodenal tissues were analyzed by a default algorithm in network visualization of MetaCore. The PPAR-α signaling pathway was the center of the signaling networks. All of these pathways are essential to the survival of cancer cells (Figure 3). Therefore, the PPAR-α signaling and lipid metabolism-related pathways were selected to investigate the mechanism of carcinogenesis in ampullary cancer.

### Extracellular matrix-related pathways in the GSEA

**Figure 4:**
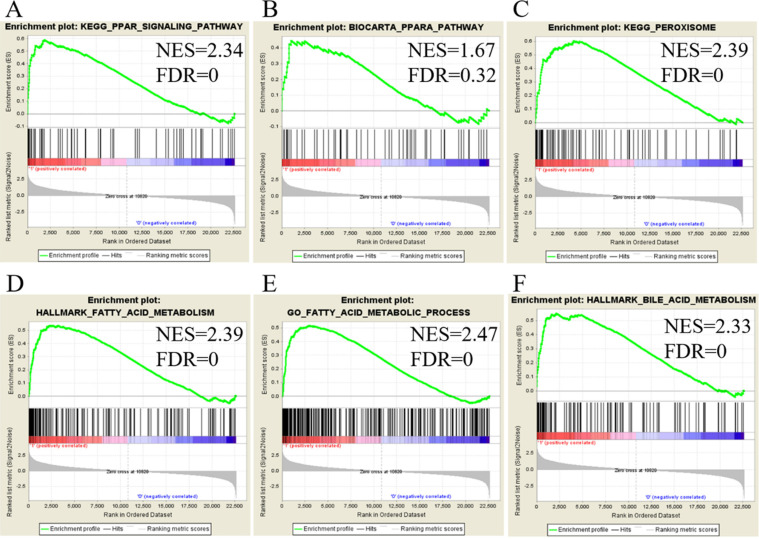
** Analysis of upregulated genes in ampullary cancer by the Kyoto Encyclopedia of Genes and Genomes (KEGG), BioCarta, Gene Ontology (GO), and Hallmark databases.** The enrichment score (y-axis) reflects the increased degree of associated genes in ampullary cancer (Y = 1 = cancer) compared to normal duodenal tissues (Y = 0 = normal). The green line indicates the evolution of the density of genes identified in the microarray dataset. The horizontal bar in a gradation of red to blue represents the rank of genes in the ordered dataset. Genes on the left side (red) were correlated with the most strongly associated ones, while genes on the right side (blue) are those with negative correlation. Each solid bar in the middle represents each gene within a gene set. A normalized enrichment score (NES) was employed to compute the density of modified genes in the microarray with random expectancies. The false discovery rate (FDR) is the estimated probability with a given NES and is represented as a false positive finding. All *p* values were < 0.001. (A) Peroxisome proliferator-activated receptor (PPAR) signaling in the KEGG. (B) PPARA signaling in BioCarta. (C) Peroxisome in the KEGG. (D) Fatty acid metabolism in Hallmark. (E) Fatty acid metabolism in GO. (F) Bile acid metabolism in Hallmark.

**Figure 5:**
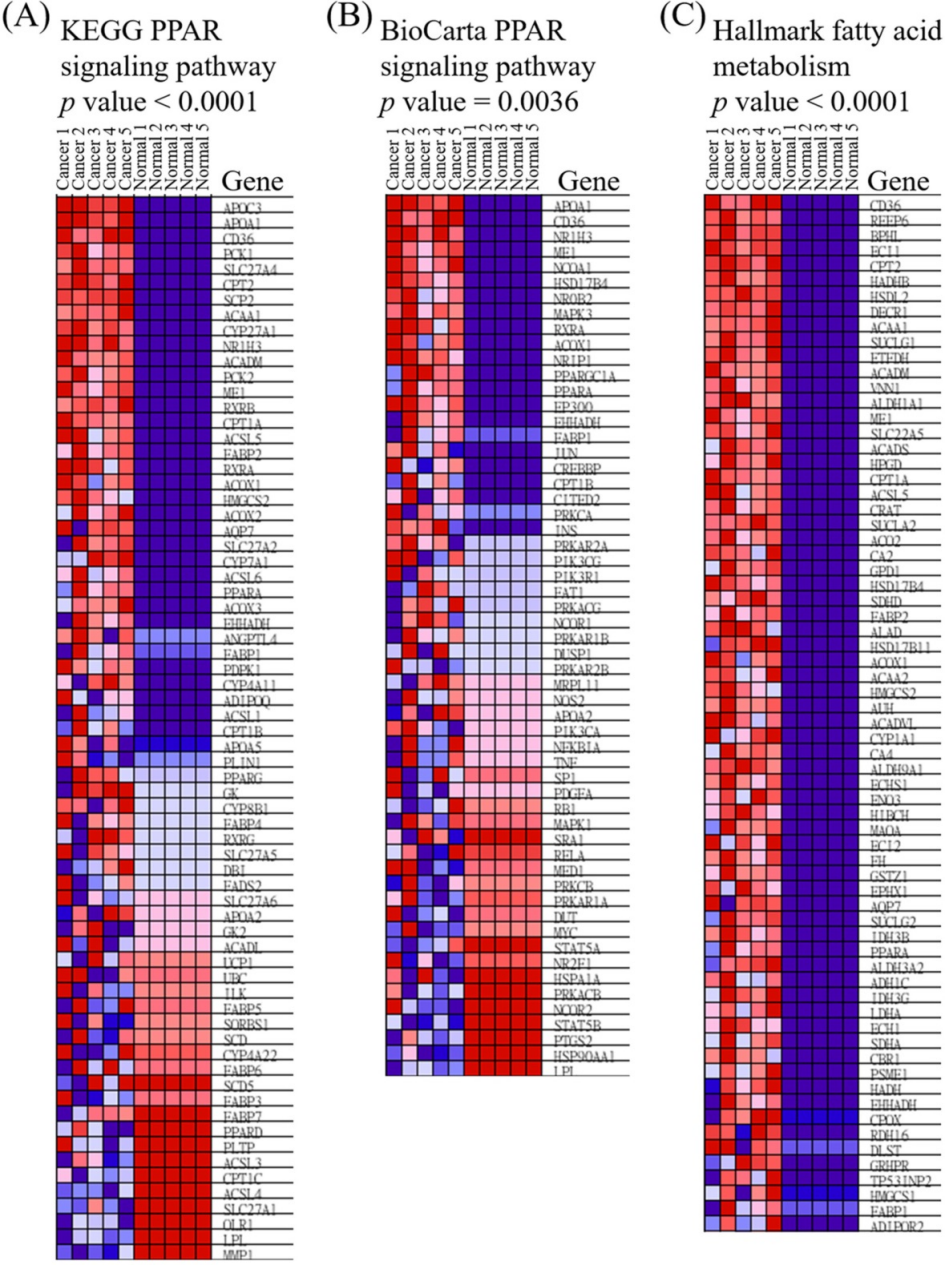
** Heatmap of expressed genes in ampullary cancer and normal duodenal tissues.** (A) Peroxisome proliferator-activated receptor (PPAR) signaling pathway in the Kyoto Encyclopedia of Genes and Genomes (KEGG). (B) PPAR in BioCarta. (C) Fatty acid metabolism in Hallmark. The color gradient matches the ranking of particular genes in ampullary cancer. Red indicates upregulation, and blue indicates downregulation.

We utilized public databases to verify the importance of targeted pathways as shown in Figure 2. DEGs in ampullary cancer were analyzed. Enriched pathways included the PPAR signaling pathway (Figure 4A, B), peroxisome-related signaling (Figure 4C), and metabolism of fatty acids (Figure 4D, E) and bile acids (Figure 4F). The analytic software included KEGG (Figure 4A, C), BioCarta (Figure 4B), Hallmark (Figure 4D, F), and GO (Figure 4E). In a comparison between ampullary cancer and corresponding normal duodenal tissues, upregulated genes are shown in red in Figure 5. Genes in ampullary cancer that were upregulated compared to those in normal duodenal tissues were analyzed using the GSEA to identify carcinogenesis-related genes. The PPAR signaling (KEGG in Figure 5A and BioCarta in Figure 5B) and lipid metabolism pathways (Hallmark in Figure 5C) were enhanced in all five ampullary cancer cases examined. Altogether, PPAR and lipid metabolism-related genes were enriched in three different datasets, suggesting their crucial roles in ampullary cancer development.

### External validation in public high-throughput databases

**Figure 6:**
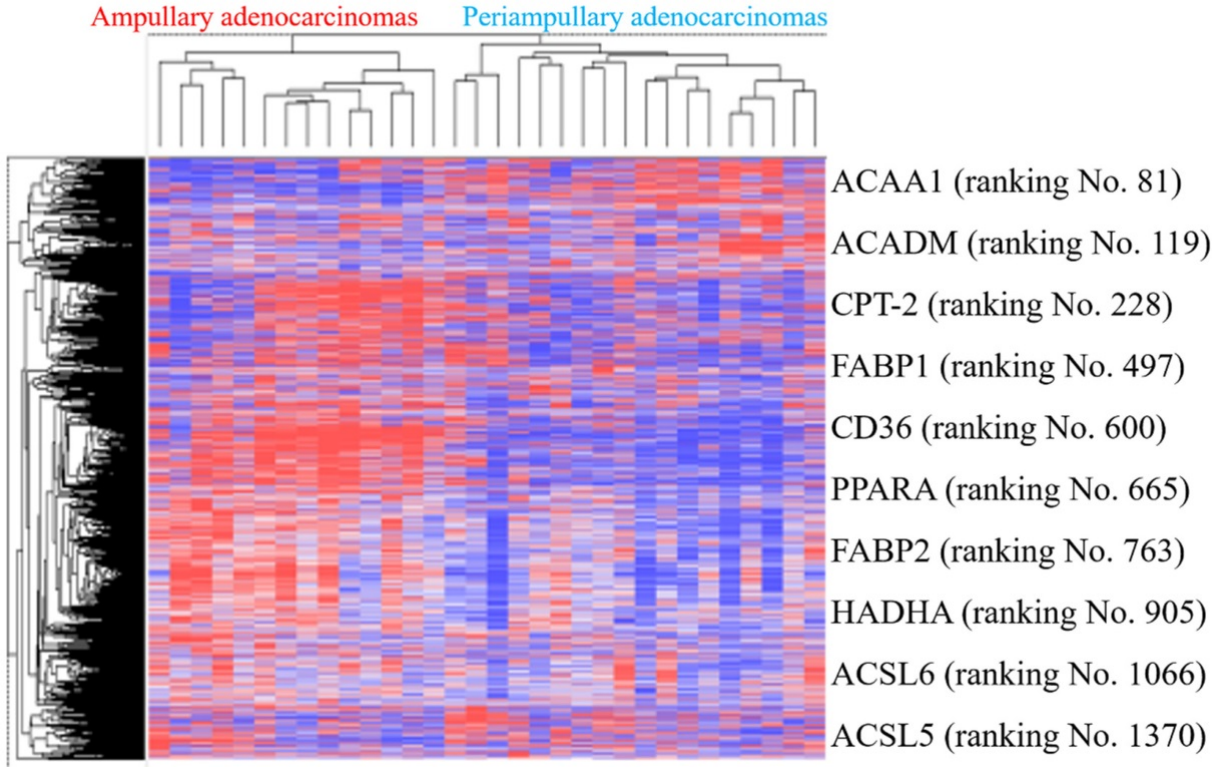
** Heatmap of expression (cancer/normal) ratios in the GSE39409 dataset.** The top highly expressed genes in ampullary cancer tissues were identified by hierarchical clustering with Pearson's coefficient. Genes in the heatmap represent the top 10% of genes with the highest correlation coefficients in ampullary adenocarcinomas compared to periampullary adenocarcinomas. Genes listed on the right side include peroxisome proliferator-activated receptor alpha (*PPARA*) signaling and lipid metabolism-related ones.

**Figure 7:**
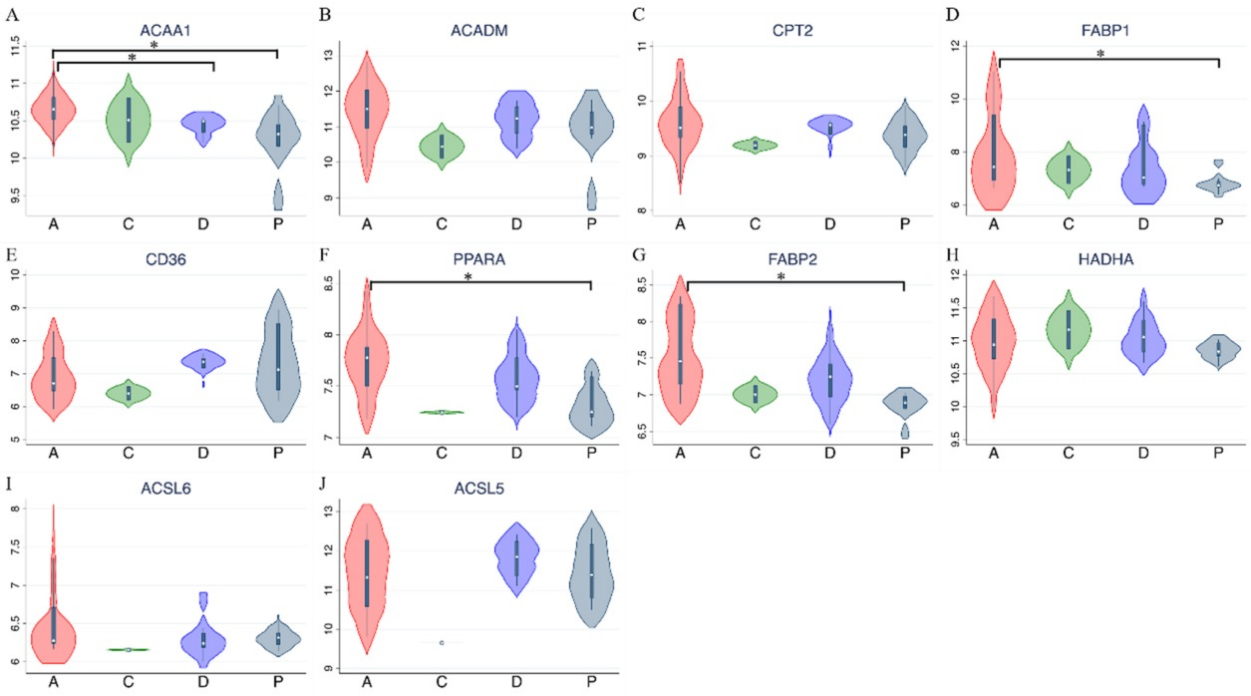
** Expression levels of target genes in the GSE39409 dataset.** Violin plots for targeted genes in peroxisome proliferator-activated receptor alpha (*PPARA*) and lipid metabolism-related pathways, including (A) *ACAA1*; (B) *ACADM*; (C) *CPT2*; (D) *FABP1*; (E) *CD36*; (F) *PPARA*; (G) *FABP2*; (H) *HADHA*; (I) *ACSL6*; (J) *ACSL5*. A, ampullary adenocarcinoma; C, extrahepatic biliary cholangiocarcinoma; D, duodenal adenocarcinoma; P, pancreatic adenocarcinoma; * *p* < 0.05 after the Bonferroni correction was applied.

PPAR and lipid metabolism-related signaling genes are highly expressed in ampullary cancer compared to normal tissues (Figures 1~4). To validate our findings and correlate them with a previous study, the GSE39409 dataset was downloaded from the National Center for Biotechnology Information Gene Expression Omnibus (NCBI GEO) database. This dataset is composed of microarray data of 32 untreated periampullary adenocarcinomas and was analyzed by the Affymetrix U133 Plus 2.0 genome array (including 14 ampullary, eight pancreatic, eight duodenal, and two extrahepatic biliary cholangiocarcinomas) 12. A heatmap was chosen to display these data due to the high dimensions, efficiency, and speed. Microarray data were categorized into two groups: patients with ampullary adenocarcinomas (*n* = 14) and patients with other periampullary adenocarcinomas (*n* = 18). The heatmap of gene expression rankings indicated that *PPARA* and lipid metabolism-related genes were highly expressed in ampullary adenocarcinomas compared to periampullary adenocarcinomas. As seen in Figure 6, ten genes associated with *PPARA* and lipid metabolism ranked in the top 5% of 54,675 detected genes in the microarray. *PPARA* and lipid metabolism-related genes can serve as important biomarkers in ampullary cancer patients. Previous research indicated that these genes may contribute to carcinogenesis 35. Expression levels of these 10 genes in the GSE39409 dataset were represented in a violin plot to demonstrate differences among ampullary adenocarcinomas, cholangiocarcinomas, and duodenal and pancreatic adenocarcinomas (Figure 7). Increased expressions of *ACAA1* (acetyl-CoA acyltransferase 1, Figure 7A), *FABP1* (fatty acid-binding protein 1, Figure 7D), *PPARA* (Figure 7F), and *FABP2* (Figure 7G) genes were seen in ampullary adenocarcinomas compared to pancreatic adenocarcinomas. Trends of higher expressions of other lipid metabolism-related genes in ampullary adenocarcinoma were also detected (*ACADM* in Figure 7B, *CPT2* in Figure 7C, *HADHA* in Figure 7H). Expressions of some genes were similar in all of these periampullary adenocarcinomas (*CD46* in Figure 7E, *ACSL6* in Figure 7I, and *ACSL5* in Figure 7J).

### Expression of lipid metabolism-related genes in ampullary cancers

A qPCR and semiquantitative RT-PCR were used to compare expressions of lipid-metabolism genes in cell lines from ampullary cancer and other cancers. Increased expressions of *PPARA1, ACAA1, FABP1,* and *FABP2* were detected in SNU-478 ampullary cancer cells compared to pancreatic cancer cells (Pan1 and MIA-Pan2) and gastric cancer cells (AGS) by qPCR (Supplementary Figure 2A-D). Expression of *PPARA* in TGBC-18 TKB ampullary cancer cells was higher in gastric cancer cells (AGS and MKN45) and colon cancer cells (HCT116) by semiquantitative RT-PCR (Supplementary Figure 2E-F). Expression of* PPARA* mRNA in ampullary cancer cell lines was higher than in other cancer cell lines. The PPAR-α protein was detected by IHC staining. Low expression of the PPAR-α protein was detected in three and high PPAR-α expression in eight of 11 samples (Supplementary Figure 3). These validated data were consistent with our bioinformatics analysis; therefore, these genes may play crucial roles in cancer development and could serve as potential biomarkers in ampullary cancer patients.

### Identification of potential inhibitory compounds with CMap and their validation in ampullary cancer cell lines

**Figure 8:**
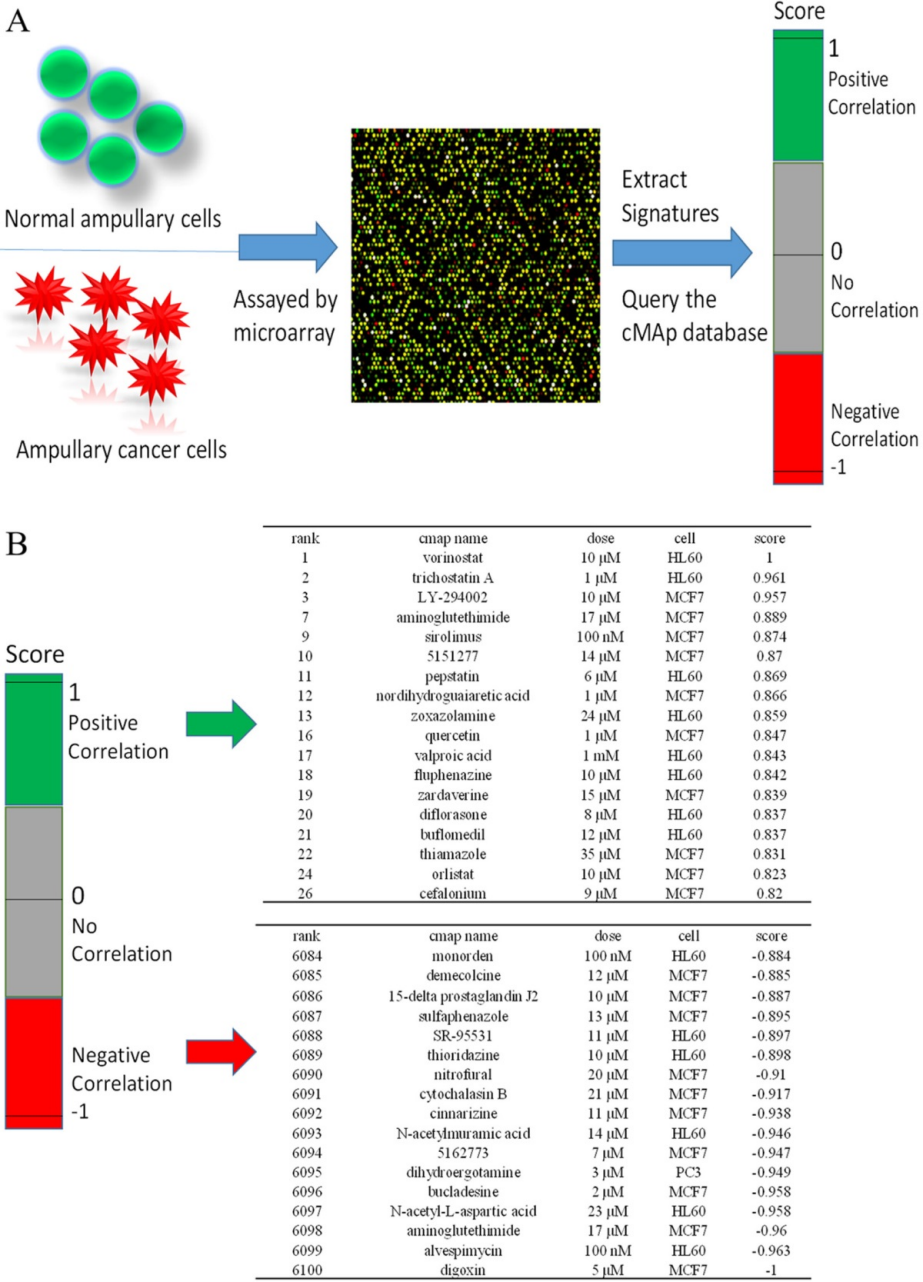
** Connectivity Map (CMap) analysis based on differential expressions of genes in the microarray.** (A) Flowchart of the study protocol. Results of the CMap analysis are represented as +1 (top, lowest potential of inhibition) to -1 (bottom, highest potential of inhibition). Drugs with a positive score are presented in green, while those with negative connectivity score are displayed in red. (B) The top 18 and the bottom 17 drugs were ranked according to their score.

We used CMap to investigate potential drugs that may inhibit upregulated genes in ampullary cancer. One of the advantages of CMap is its ability to predict the efficacy of drugs based on transcriptional expressions of genes. We collected information from 1309 chemicals in total, for which more than 7000 treatments with different dosages were provided in CMap. Detailed drug candidates are listed in Supplementary Table 4. Molecules with the most negative correlations had the highest potential for therapeutic effects against ampullary cancer (Figure 8A), including digoxin (medication for atrial fibrillation and heart failure), alvespimycin (17-dimethylaminoethylamino-17-demethoxygeldanamycin, an analogue of the antineoplastic drug, geldanamycin), aminoglutethimide (blocks the formation of adrenal steroids and estrogen), *N*-acetyl-L-aspartic acid (for synthesis of neurotransmitters), bucladesine (a cyclic nucleotide derivative), dihydroergotamine (a vasoconstrictor), compound 5162773 (trichostatin A, a reversible inhibitor of histone deacetylase (HDAC)), *N*-acetylmuramic acid (part of bacterial cell walls), cinnarizine (an antihistamine), and cytochalasin B (blocks formation of contractile microfilaments) (Figure 8B).

The cytotoxic abilities of alvespimycin, cytochalasin B, and trichostatin A were examined by a cell proliferation assay (Supplementary Figure 4). Alvespimycin suppressed the growth of SNU-478 and TGBC-18 TKB cells with a higher dose of 10 μM and trichostatin A with a lower dose of 1 μM. Cytochalasin B only had a weak ability as a cytostatic compound. IC_50_ levels are shown in Supplementary Figure 5. After treatment for 48 h, only trichostatin A had a lower IC_50_ for SNU-478 (0.047 μM) and TGBC-18 TKB cells (0.159 μM). Alvespimycin and cytochalasin B had extremely high IC_50_ levels.

## Discussion

Ampullary adenocarcinomas are a rare periampullary cancer with 5-year cancer-specific survival rates of 30%~50% 36-38. Adjuvant chemoradiotherapy fails to improve survival 6. Survival rates of ampullary cancer patients differ from those with other periampullary cancers, which implies distinct carcinogenic mechanisms 39. In the present study, we utilized a cDNA microarray from five pairs of ampullary cancer and normal duodenal tissues to study the carcinogenic mechanism. After the bioinformatic analyses with KEGG, BioCarta, Hallmark, Reactome, NCBI GEO, and MetaCore from the GeneGO databases, PPAR-α and lipid metabolism-related pathways were found to be the most critical signaling pathways in ampullary cancer. Under the CMap investigation, potential drugs targeting cancer cells with upregulated genes from microarray results were identified. These drugs could potentially be effective in treating ampullary cancer in the future.

Several biomarkers for prognostic prediction of ampullary cancer patients were identified in previous studies 7-9. However, the techniques used in those studies were limited and might not be representative of the cancer-related pathways. Further, it is difficult to identify novel biomarkers by tissue microarrays because of cancer heterogeneity 40. To address this problem, we collected fresh ampullary cancer and normal duodenal tissues. Holistic high-throughput technology with a cDNA microarray was performed to detect whole gene expressions. For the GSEA of microarray data, KEGG 41, BioCarta 42, Hallmark 43, Reactome 44, NCBI GEO 45, and MetaCore from GO 46 are popular databases. These platforms were seldom applied in studies of ampullary cancer; however, we utilized all of these bioinformatics approaches to identify potential pathways in the carcinogenesis of ampullary cancer (Supplementary Figure 1). Our analysis demonstrated that PPAR and lipid metabolism-related pathways (Figure 1), such as “Regulation of lipid metabolism PPAR regulation of lipid metabolism” and “Regulation of lipid metabolism RXR-dependent regulation of lipid metabolism via PPAR, RAR, and VDR” (Figure 2) were important in ampullary cancer. Further analyses with KEGG, BioCarta, Hallmark, Reactome, and GO also indicated that *PPARA* and lipid metabolism-related genes were enhanced in ampullary cancer (Figures 3~5). Due to the limited sample size of our study, the public database GSE39409 from NCBI GEO was selected to provide external validation. A heatmap study of GSE39409 further supported *PPARA* and lipid metabolism-related genes being highly correlated with carcinogenesis of ampullary adenocarcinomas, but not other periampullary adenocarcinomas (Figures 6, 7). We also found *PPARA* expression in cell lines and clinical samples from ampullary cancer (Supplementary Figure 2, 3). These findings will contribute to a new understanding of the molecular mechanisms of ampullary cancer.

PPAR proteins consist of three types: PPAR-α, PPAR-β (also called PPAR-δ), and PPAR-γ. There are some differences in tissue distributions, ligands, and target specificities among these family members 47. PPAR-α is highly expressed in tissues with intensive fatty acid oxidation, such as the liver, heart, muscles, kidneys, and cells of arterial walls. One group of its physiological ligands is long-chain fatty acids. RXR-α and PPAR-α form a heterodimer that acts on peroxisome proliferator response elements and controls expressions of numerous genes in fatty acid oxidation 48. PPAR-α regulates expressions of major genes in fatty acid oxidation and represents a key point of lipid metabolism 49. Downstream genes of PPAR-α include acyl-CoA synthetase long-chain family member 1/5 (*ACSL1/5*), mitochondrial medium-chain acyl-CoA dehydrogenase (*ACADM*), mitochondrial enoyl-CoA hydratase/3-hydroxy acyl-CoA dehydrogenase (*HADHA*), and *ACAA1*. PPAR-α also controls expressions of genes which enable cellular transport of fatty acids and their derivatives, such as cluster of differentiation 36 (*CD36*), carnitine palmitoyltransferase 1A (*CPT1A*), 1B (*CPT1B*), and 2 (*CPT2*), and *FABP2* 50. PPAR-α was reported to play important roles in various cellular activities, including proliferation, metabolism, homeostasis, and tumorigenesis 51. PPAR-α promotes proliferation of breast cancer cells 52. Inhibition of *PPARA* gene induces immunogenic death of proliferating chronic lymphocytic leukemia cells 53. On the other hand, PPAR-α agonists inhibit the growth of gliomas and cancers of the colon, lung, and ovaries 17. Thus, the function of PPAR-α in tumorigenesis is still controversial. In the present study, cDNA microarrays from five paired ampullary cancer and normal duodenal tissues were first studied, and then the GSE39409 dataset was utilized for validation. Upregulation of PPAR-α signaling was detected in both analyses. Expressions of *PPARA* mRNA and PPAR-α protein were also detected in clinical samples and cell lines of ampullary cancer. Our results implied that PPAR-α may promote the carcinogenesis of ampullary cancer.

Since traditional chemoradiotherapy fails to improve the survival of ampullary cancer patients 5, we searched potential FDA-approved drugs for ampullary cancer using CMap software. In CMap, alterations of gene expressions were recorded in cell lines treated with potential bioactive molecules (Figure 8A). The union of upregulated genes is positively correlated with certain drugs, and those drugs are presumed to be cancer promoters. On the contrary, upregulated genes are negatively correlated with other drugs, and those drugs are assumed to be cancer inhibitors. In our results, the top ten drugs with negative correlations included digoxin, alvespimycin, aminoglutethimide, *N*-acetyl-L-aspartic acid, bucladesine, dihydroergotamine, compound 5162773, *N*-acetylmuramic acid, cinnarizine, and cytochalasin B (Figure 8B). Alvespimycin binds to heat shock protein 90 (Hsp90), leading to its degradation and suppression of cell cycle progression 54. The HDAC inhibitor, compound 5162773 (trichostatin A), suppresses acetylation of chromosomes and restrains gene expressions in cancer cells 55. Cytochalasin B is a microfilament-directed agent with cytotoxic ability against lung carcinomas and melanomas 56. We tested the cytotoxic abilities of alvespimycin, cytochalasin B, and trichostatin A in ampullary cancer cell lines (Supplementary Figure 4, 5). Trichostatin A had the strongest ability to suppress proliferation with the lowest IC_50_. The drug and/or its derivatives are potential drugs that could improve the survival status of ampullary cancer patients.

In conclusion, the present study combined a cDNA microarray in our institution and the online GSE39409 database to investigate carcinogenic mechanisms of ampullary cancer. Our results identified *PPARA* and lipid metabolism-related genes as being upregulated in ampullary cancer. These genes are conceivable biomarkers for diagnosis of ampullary cancer and also provide hints to identify potential antineoplastic agents. According to results of CMap and cellular experiments, the HDAC inhibitor, trichostatin A, is a potential drug for ampullary cancer patients. Thus, the present study provides a theoretical basis for further research.

## Supplementary Material

Supplementary figures and tables.

## Figures and Tables

**Table 1:** Kyoto Encyclopedia of Genes and Genomes (KEGG) pathway enrichment analysis in ampullary cancer microarray by the Database for Annotation, Visualization and Integrated Discovery (DAVID). This table contains only pathways with *p* values of < 0.0001

KEGG_PATHWAY	*p* value	Genes
PPAR signaling pathway	2.05E-11	*ME1, ACOX2, ACOX1, PPARA, ACADM, CPT2, AQP7, PCK2, PCK1, APOA1, CD36, CYP27A1, HMGCS2, CYP7A1, APOC3, FABP1, FABP2, GK, SCP2, SLC27A2, ACSL6, ACSL5, ACAA1, NR1H3, SLC27A4*
Retinol metabolism	2.78E-10	*CYP3A4, CYP3A5, CYP3A7, CYP1A1, CYP2C19, CYP2C18, CYP2C9, CYP2B6, ADH6, RDH5, ALDH1A1, UGT1A6, UGT1A8, DGAT1, LRAT, DGAT2, ADH4, UGT2B11, UGT2A3, UGT2B10, UGT2B7, RETSAT*
Drug metabolism	4.65E-09	*CYP3A4, GSTA2, CYP3A5, CYP3A7, CYP2C19, GSTA5, CYP2C18, CYP2C9, CYP2B6, MAOA, MAOB, CYP2D6, ADH6, FMO4, FMO5, UGT1A6, UGT1A8, ADH4, UGT2B11, UGT2B10, UGT2A3, UGT2B7*
Metabolism of xenobiotics by cytochrome P450	1.61E-08	*CYP3A4, GSTA2, CYP3A5, CYP3A7, CYP1A1, CYP2C19, GSTA5, CYP2C18, CYP2C9, CYP2B6, CYP2S1, ADH6, DHDH, AKR1C3, UGT1A6, UGT1A8, ADH4, UGT2B11, UGT2B10, UGT2A3, UGT2B7*
Steroid hormone biosynthesis	2.24E-06	*CYP3A4, CYP3A5, CYP3A7, CYP1A1, HSD17B2, AKR1C3, UGT1A6, UGT1A8, CYP7A1, UGT2B11, HSD11B2, HSD17B3, UGT2B10, SULT1E1, UGT2A3, UGT2B7*
Fatty acid metabolism	2.27E-06	*ACAA2, ACOX1, ACADSB, CPT2, ACADM, ACADS, ADH4, ADH6, ACAT1, HADHA, ACSL6, ACAA1, ACSL5, HADHB*
Linoleic acid metabolism	1.34E-05	*CYP3A4, CYP3A5, CYP2J2, AKR1B15, CYP3A7, CYP2C19, CYP2C18, CYP2C9, AKR1B10, PLA2G12B, PLA2G1B, PLA2G2A*
Arachidonic acid metabolism	2.79E-05	*CYP2J2, CYP2C19, CYP2C18, CYP2C9, CYP2B6, EPHX2, GGT1, AKR1C3, CBR1, PLA2G12B, GPX3, PLA2G1B, PLA2G2A, CYP4F3, CYP4F2*
Arginine and proline metabolism	6.70E-05	*ACY1, NAGS, OTC, MAOA, SAT2, MAOB, AGMAT, CPS1, CKMT1A, GLS, ARG2, PRODH2, OAT, PRODH*

Abbreviations: PPAR, peroxisome proliferator-activated receptor.
