# Supplementary material for: Upregulation of peroxisome proliferator-activated receptor-α and the lipid metabolism pathway promotes carcinogenesis of ampullary cancer
Source: Int J Med Sci. 2021 Jan 1;18(1):256–69. doi: 10.7150/ijms.48123 (PMC7738964; doi:10.7150/ijms.48123)
Supplement: Supplementary file 1 — Supplementary figures and tables. [file ijmsv18p0256s1.pdf]

Upregulation of peroxisome proliferator-activated receptor-α and the lipid metabolism pathway promotes carcinogenesis of ampullary cancer

Chih-Yang Wang, Ying-Jui Chao, Yi-Ling Chen, Tzu-Wen Wang, Nam Nhut Phan, Hui-Ping Hsu, Yan-Shen Shan, Ming-Derg Lai

Supplementary Table 1. Demographics and clinical outcomes of five patients with ampullary cancer

Age (years)	Sex	Staging	Tumor size (cm)	Morphology	Differentiation	Recurrence	Time to recurrence (months)	Condition	Time of survival/ expired (months)
51	F	T2N0, stage Ib	2×1×1	Polypoid	Unknown	No	--	Survived	193
58	F	T2N0, stage Ib	2.4×1.5 ×0.6	Mixed	Good	Yes	14	Expired	17
68	M	T3N0, stage IIA	4.5×3.5 ×1.2	Polypoid	Good	No	--	Survived	162
66	M	T3N0, stage IIA	1×1×0.8	Ulcerative	Good	Yes	64	Expired	227
60	M	T3N0, stage IIA	2×1.8×1	Mixed	Moderate	Yes	5.6	Expired	16.7

Supplementary Table 2. Kyoto Encyclopedia of Genes and Genomes (KEGG) pathway enrichment analysis of an ampullary cancer microarray using the Database for Annotation, Visualization and Integrated Discovery (DAVID). This table contains only pathways with *p* values that ranged 0.0001~0.05.

KEGG Pathway	*p* value	Genes
Pentose and glucuronate interconversions	1.50E-04	*UGT1A6, CRYL1, UGT1A8, AKR1B1, UGT2B11, UGT2A3, UGT2B10, UGT2B7, XYLB*
Drug metabolism	1.63E-04	*CYP3A4, XDH, UGT1A6, CYP3A5, CES2, CYP3A7, UGT1A8, NAT2, UGT2B11, DPYD, UGT2A3, UGT2B10, UGT2B7*
Maturity-onset diabetes of the young	2.43E-04	*HNF1A, HNF4A, SLC2A2, PKLR, NEUROD1, HNF4G, PDX1, NR5A2, NKX2-2*
Starch and sucrose metabolism	6.03E-04	*GBA3, UGT1A6, G6PC, UGT1A8, ENPP3, MGAM, SI, UGT2B11, TREH, UGT2A3, UGT2B10, UGT2B7*
Valine, leucine and isoleucine degradation	8.94E-04	*ACAA2, ALDH6A1, ACADSB, ACADM, HMGCS2, ACADS, ABAT, ACAT1, HADHA, ACAA1, HADHB*
Alanine, aspartate and glutamate metabolism	1.21E-03	*ASPA, ACY3, GLS, ABAT, GPT, AGXT2, CPS1, AGXT, DDO*
Glycine, serine and threonine metabolism	1.21E-03	*GLYCTK, SHMT1, CTH, MOA, AMT, BHMT, MAOB, AGXT2, AGXT*
Fructose and mannose metabolism	2.30E-03	*KHK, AKR1B15, MPI, PFKFB4, AKR1B10, PFKFB2, ALDOC, ALDOB, AKR1B1, FBP1*
Glycolysis / Gluconeogenesis	3.20E-03	*G6PC, GALM, ADH4, ALDOC, PKLR, ALDOB, FBP1, ADH6, PGAM2, ACSS2, PCK2, PCK1*
ABC transporters	3.48E-03	*ABCA8, ABCG8, ABCG5, ABCB1, ABCC2, ABCA4, ABCC8, ABCA5, ABCG2, ABCC6*
Androgen and estrogen metabolism	4.04E-03	*UGT1A6, UGT1A8, HSD17B2, UGT2B11, HSD11B2, HSD17B3, UGT2A3, UGT2B10, SULT1E1, UGT2B7*
Nitrogen metabolism	4.71E-03	*CTH, CA7, AMT, GLS, CA4, CA2, CPS1*
Sulfur metabolism	8.22E-03	*CHST13, SULT1A2, SULT1A4, SULT1E1, BPNT1*
Histidine metabolism	1.54E-02	*DDC, ASPA, CNDP1, ACY3, MAOA, MAOB, FTCD*

Pantothenate and CoA biosynthesis	1.91E-02	*PANK3, PANK1, ENPP3, VNN1, DPYD*
Tryptophan metabolism	2.23E-02	*DDC, CYP1A1, MAOA, MAOB, HAAO, CAT, ACAT1, HADHA*
Primary bile acid biosynthesis	2.40E-02	*ACOX2, CYP27A1, CYP7A1, PIWIL2, SCP2*
Propanoate metabolism	2.44E-02	*ALDH6A1, ACADM, SUCLG1, ABAT, ACSS2, ACAT1, HADHA*
Porphyrin and chlorophyll metabolism	2.81E-02	*UGT1A6, ALAD, UGT1A8, HMOX1, UGT2B11, UGT2A3, UGT2B10, UGT2B7*
Renin-angiotensin system	2.97E-02	*ACE, ACE2, MME, ANPEP, ENPEP*
Ascorbate and aldarate metabolism	2.97E-02	*UGT1A6, UGT1A8, UGT2B11, UGT2A3, UGT2B10, UGT2B7*
Butanoate metabolism	3.22E-02	*AKR1B15, HMGCS2, ACADS, AKR1B10, ABAT, BDH2, ACAT1, HADHA*
Galactose metabolism	3.52E-02	*GALK1, G6PC, AKR1B1, MGAM, GALT, LCT*
Glycerolipid metabolism	3.99E-02	*DGKA, GLYCTK, PNLIPRP2, DGAT1, DGAT2, DAK, AKR1B1, GK*

Supplementary Table 3. Functional enrichment of Gene Ontology (GO) biological processes analysis in a cDNA microarray of ampullary adenocarcinomas by the Database for Annotation, Visualization and Integrated Discovery (DAVID)

GO term	*p* value	Genes
Digestion	1.35E-13	*SLC15A1, SLC5A1, PRSS1, VIPR1, SCTR, APOA4, VDR, SCT, UGT1A6, ACE, UGT1A8, PLA2G1B, NPC1L1, SOAT2, MOGAT2, PNLIPRP2, SULT2A1, PPARGC1A, ABCG8, SSTR2, ABCG5, AKR1B15, SSTR1, AKR1B10, MEP1A, ACE2, MEP1B, GHRL, FABP1, FABP2, SST*
Fatty acid metabolic process	1.99E-13	*ACOX2, HACL1, ACOX1, PPARA, ACADSB, CYP2J2, CPT2, HNF1A, EDN2, ECHDC2, ACSF3, ACSF2, HADHA, HADHB, AKR1C3, PECR, CRYL1, ACOT11, PLA2G1B, ETFDH, ELOVL7, BDH2, ACSL6, ACSL5, ACAA2, PTGR1, ACADM, LIPA, ACADS, EPHX2, DECR1, FADS6, PPARGC1A, CYP4F8, CYP4F3, FABP2, CYP4F2, SLC27A3, HPGD, SLC27A2, CROT, SLC27A4, ACAA1*
Oxidation reduction	2.23E-13	*ALDH8A1, ACOX2, CYP3A4, ACOX1, CYP3A5, HTATIP2, CYP3A7, CYP2J2, CYP2C19, CYP2C18, CYP2S1, CYP2D6, AKR1C3, GSR, PECR, MSRA, HMOX1, CYP7A1, GPX3, AKR7A2, AKR7A3, PIWIL2, CAT, ALDH6A1, ACADM, CYP1A1, ACADS, CYB5A, DECR1, DHRS7C, PPARGC1A, DDO, POR, DHDH, RDH5, DHRS1, AKR1B15, CYP27A1, AKR1B10, CYBRD1, HSD11B2, PRODH, HSD17B11, ME1, XDH, ACADSB, HSD17B2, CYP2B6, ADH6, HADHA, ALDH1A1, FMO4, FMO5, CBR1, ADH4, ETFDH, HAAO, BDH2, HSD17B3, GLRX, AKR7L, GPD1, PAOX, PTGR1, CYP2C9, MAOA, MAOB, DHRS11, CYP4F11, CYP4F12, CYP4V2, PHYH, FADS6, IDH3A, IYD, CYP4B1, SDHA, ADI1, CYP4F8, AKR1B1, PRODH2, CYP4F3, DPYD, CYP4F2, ACAD11, HPGD, RETSAT, CRYZL1*
Carboxylic acid transport	1.55E-11	*SLC36A1, PPARA, CPT2, HNF1A, SLC16A10, DRD4, SLC7A9, SLC19A1, AGXT, SLC7A7, MIP, SLC25A20, SLC23A1, ACE, PLIN2, SLC1A7, SLC22A4, PLA2G1B, SLC22A5, SLC1A1, SLC6A12, SLC3A1, SLC10A2, SLC6A19, CD36, SLC26A8, SLC6A5, FABP1, FABP2, SLC25A15, PDZK1, SLC46A1, CROT, SLC27A4*
Organic acid transport	1.89E-11	*SLC36A1, PPARA, CPT2, HNF1A, SLC16A10, DRD4, SLC7A9, SLC19A1, AGXT, SLC7A7, MIP, SLC25A20, SLC23A1, ACE, PLIN2, SLC1A7, SLC22A4, PLA2G1B, SLC22A5, SLC1A1, SLC6A12, SLC3A1, SLC10A2, SLC6A19, CD36, SLC26A8,*

		SLC6A5, FABP1, FABP2, SLC25A15, PDZK1, SLC46A1, CROT, SLC27A4
Lipid transport	2.39E-10	*PPARA, CPT2, MSR1, HNF1A, ATP10A, DRD4, APOC2, ABCA4, APOA4, SLC25A20, APOB, ACE, APOA1, PLIN2, APOC3, APOH, PLA2G1B, NPC1L1, SCARB1, GLTPD2, APOM, SOAT2, MTTP, ABCG8, ABCG5, CD36, FABP1, FABP2, SCP2, CROT, APOL5, SLC27A4*
Lipid localization	4.48E-10	*PPARA, CPT2, MSR1, HNF1A, ATP10A, DRD4, APOC2, ABCA4, ACVR1C, APOA4, SLC25A20, APOB, ACE, APOA1, PLIN2, APOC3, APOH, PLA2G1B, NPC1L1, SCARB1, GLTPD2, APOM, SOAT2, MTTP, ABCG8, ABCG5, CD36, FABP1, FABP2, SCP2, CROT, APOL5, SLC27A4*
Steroid metabolic process	4.87E-10	*HSD17B11, CYP3A4, ACOX2, CYP3A5, HNF1A, HSD17B2, APOA4, SULT4A1, UGT1A6, APOB, APOA1, UGT1A8, CYP7A1, APOC3, NPC1L1, SULT1A2, PIWIL2, HSD17B3, SCARB1, CAT, SULT1E1, SULT1A4, SOAT2, ACAA2, LIPA, SULT2A1, CYP1A1, NR0B2, GBA2, G6PC, NR1I2, AKR1B15, HMGCS2, CYP27A1, SULT1B1, AKR1B10, UGT2B11, HSD11B2, NR5A2, SCP2*
Triglyceride metabolic process	1.02E-09	*MOGAT2, PNLIPRP2, APOC2, CPS1, PCK1, MTTP, APOA4, APOB, G6PC, DGAT1, DGAT2, APOC3, AGPAT9, APOH, SLC22A4, CAT, NR1H3*
Regulation of hormone levels	2.98E-09	*ALDH8A1, HSD17B11, EDN3, HNF1A, FAM3B, PDX1, FKBP1B, PCSK2, UGT1A6, PCSK1, ACE, APOA1, UGT1A8, ADH4, SCARB1, SULT1E1, PCSK5, SHBG, CYP1A1, TBX3, DDO, DGAT1, LRAT, GHRH, SULT1B1, UGT2B11, ACE2, NEUROD1, HSD11B2, GHRL, SMPD3, RETSAT*
Organic ether metabolic process	4.30E-09	*MOGAT2, PNLIPRP2, CYP1A1, APOC2, CPS1, PCK1, MTTP, APOA4, APOB, G6PC, DGAT1, DGAT2, APOC3, AGPAT9, APOH, SLC22A4, CAT, NR1H3*
Lipid catabolic process	5.68E-09	*HSD17B11, ACOX2, ENPP6, HACL1, ACOX1, CPT2, APOC2, HADHA, HADHB, APOA4, PLCB3, APOB, SMPDL3B, SMPDL3A, CYP7A1, PLA2G12B, APOC3, ETFDH, PLA2G1B, SCARB1, BDH2, PLD1, ACADM, LIPA, PNLIPRP2, SULT2A1, ACADS, DECR1, CPS1, GBA2, PLA2G2A, SMPD3, ACAA1*
Cellular lipid catabolic process	8.88E-09	*ACOX2, HACL1, ACOX1, PLD1, ACADM, CPT2, ACADS, DECR1, CPS1, HADHA, HADHB, GBA2, PLCB3, APOB, SMPDL3B, SMPDL3A, APOC3, ETFDH, BDH2, SMPD3, ACAA1*

Acylglycerol metabolic process	9.19E-09	*MOGAT2, PNLIPRP2, APOC2, CPS1, PCK1, MTTP, APOA4, APOB, G6PC, DGAT1, DGAT2, APOC3, AGPAT9, APOH, SLC22A4, CAT, NR1H3*
Neutral lipid metabolic process	1.28E-08	*MOGAT2, PNLIPRP2, APOC2, CPS1, PCK1, MTTP, APOA4, APOB, G6PC, DGAT1, DGAT2, APOC3, AGPAT9, APOH, SLC22A4, CAT, NR1H3*
Sterol homeostasis	1.33E-08	*SOAT2, APOC2, MTTP, ABCG8, APOA4, APOB, G6PC, ABCG5, MALL, APOA1, APOC3, NPC1L1, SCARB1, NR5A2, APOM*
Cholesterol homeostasis	1.33E-08	*SOAT2, APOC2, MTTP, ABCG8, APOA4, APOB, G6PC, ABCG5, MALL, APOA1, APOC3, NPC1L1, SCARB1, NR5A2, APOM*
Glycerol ether metabolic process	1.76E-08	*MOGAT2, PNLIPRP2, APOC2, CPS1, PCK1, MTTP, APOA4, APOB, G6PC, DGAT1, DGAT2, APOC3, AGPAT9, APOH, SLC22A4, CAT, NR1H3*
Fatty acid transport	1.96E-08	*PPARA, SLC25A20, ACE, CPT2, CD36, HNF1A, PLIN2, DRD4, PLA2G1B, FABP1, FABP2, CROT, SLC27A4*
Cofactor metabolic process	3.23E-08	*MOCOS, ME1, ALAD, HNF1A, ALDOB, GGT1, PDSS1, GCH1, TPK1, UGT1A6, GSR, CBR1, UGT1A8, HMOX1, ADH4, GPX3, HAAO, VNN1, ACSL6, MOCS1, GPD1, SHMT1, ACO2, CYP1A1, SUCLG1, FTCD, IDH3A, SDHA, COQ4, CTH, PANK3, HNF4A, PANK1, QPRT, CRYZL1*
Sterol transport	8.51E-08	*SOAT2, HNF1A, MSR1, APOC2, ABCG8, APOA4, APOB, ABCG5, CD36, APOA1, APOC3, NPC1L1, SCARB1, APOM*
Cholesterol transport	8.51E-08	*SOAT2, HNF1A, MSR1, APOC2, ABCG8, APOA4, APOB, ABCG5, CD36, APOA1, APOC3, NPC1L1, SCARB1, APOM*
Lipid homeostasis	1.29E-07	*SOAT2, APOC2, MTTP, ABCG8, APOA4, APOB, G6PC, ABCG5, APOA1, MALL, HNF4A, APOC3, NPC1L1, SCARB1, NR5A2, APOM*
Response to toxin	2.86E-07	*DDC, TRPM6, PTGR1, CYP1A1, SLC6A4, EPHX2, BPHL, GUCY2C, AQP10, BAK1, SLC23A1, CDKN1A, PEBP1, HTR1D, PDZD3, MT1H, MPST*
Secretion	3.52E-07	*EDN3, SLC22A18, HNF1A, TRPV1, FAM3B, DRD4, NR3C2, CCL8, AQP7, PDX1, TPD52, AMN, AGXT, FKBP1B, AQP3, STXBP5L, SCTR, SCT, ACE, HMOX1, KCNK5, PLA2G1B, SLC22A4, GUCA2B, PCSK5, SCG2, SCAMP5, TBX3, BAIAP3, ABCG8, SLC26A3, ABCG5, TRIM36, DGAT1, GHRH, SCIN, GHRL, NEUROD1, CA2, DOC2B, SMPD3, MON1A*
Chemical homeostasis	4.52E-07	*JPH4, HNF1A, GRIK1, EDN2, GNA11, GRIK3, F2RL1, PDX1, APOA4, BAK1, APOB, APOA1, MALL, GRIN2B, AQP11, HMOX1,*

		RGN, NPC1L1, CHRNA7, CHRFAM7A, APOM, OTC, MLXIPL, GRIN2A, SLC9A3R1, PPARGC1A, CCR9, G6PC, HNF4A, GHRH, GHRL, MON1A, CALCR, PRKCZ, CCK, SLC37A4, DRD4, NR3C2, APOC2, FKBP1B, VDR, APOC3, SCARB1, SLC39A4, MT1H, GAL3ST1, SOAT2, EPHX2, ATP1A4, AFG3L2, CPS1, CCL15, PCK1, MTTP, ABCG8, ABCG5, ADRA1B, NEUROD1, NR5A2, SCARA5, IL2
Monocarboxylic acid transport	6.65E-07	*PPARA, HNF1A, CPT2, DRD4, SLC10A2, MIP, SLC25A20, ACE, CD36, PLIN2, PLA2G1B, FABP1, FABP2, CROT, SLC27A4*
Lipoprotein particle clearance	1.15E-06	*APOB, MSR1, CD36, APOA1, HMOX1, APOC3, APOC2, SCARB1, APOM*
Intestinal absorption	1.15E-06	*ABCG8, VDR, SOAT2, MOGAT2, ABCG5, SLC5A1, NPC1L1, FABP1, FABP2*
Lipid oxidation	1.38E-06	*ACOX2, ACOX1, HACL1, CPT2, ACADM, ACADS, DECR1, PPARGC1A, HADHA, HADHB, ETFDH, BDH2, ACAA1*
Fatty acid oxidation	1.38E-06	*ACOX2, ACOX1, HACL1, CPT2, ACADM, ACADS, DECR1, PPARGC1A, HADHA, HADHB, ETFDH, BDH2, ACAA1*
Organic acid catabolic process	1.62E-06	*ACOX2, HACL1, SHMT1, ALDH6A1, ACOX1, ACADM, CPT2, SULT2A1, ACADS, AMT, DECR1, TAT, DDO, HADHA, HADHB, ASPA, GLS, ETFDH, PRODH2, BDH2, ACAA1, PRODH*
Carboxylic acid catabolic process	1.62E-06	*ACOX2, HACL1, SHMT1, ALDH6A1, ACOX1, ACADM, CPT2, SULT2A1, ACADS, AMT, DECR1, TAT, DDO, HADHA, HADHB, ASPA, GLS, ETFDH, PRODH2, BDH2, ACAA1, PRODH*
Fatty acid beta-oxidation	2.28E-06	*ACOX2, ACOX1, CPT2, ACADM, ACADS, ETFDH, BDH2, DECR1, HADHA, ACAA1, HADHB*
Ion transport	2.31E-06	*KCNJ16, SLC36A1, JPH4, SLC5A4, SLC22A18, CPT2, GRIK1, GABRB2, TRPV1, SLC20A2, SLC5A1, GRIK3, KCNJ13, KCNK10, SLC25A20, SLC23A1, GRIN2B, AQP11, KCNK5, SLC22A4, CHRNA7, ANO4, SLC22A5, TRPV6, SLC4A4, CHRFAM7A, SLC1A1, ANO9, GABRG1, SGK1, TRPM6, CLCA1, GRIN2A, CNGA1, SLC26A3, SLC26A8, CATSPER2, CATSPER3, CLIC5, SLC41A2, KCNH6, AKAP7, SLC30A10, MPST, CAV2, SLC39A14, ORAI1, ENPP3, RHBG, CCL8, KCNA3, KCNJ3, FKBP1B, BEST4, VDR, TMEM37, SLCO1A2, SLC4A7, SLC39A5, SLC39A4, HCN4, CAMK2A, SLC31A1, GABRA1, SLCO4C1, ATP1A4, ATP1A1, SLC10A2, P2RX6, KCNJ6, SLC17A4, SLC13A2, KCTD16, PDZK1, SCARA5, ABCC8, PDZD3, SLC5A11, SLC5A12*

Coenzyme metabolic process	2.89E-06	*ME1, MOCOS, ALDOB, GGT1, PDSS1, GCH1, TPK1, GSR, GPX3, HAAO, VNN1, ACSL6, MOCS1, SHMT1, GPD1, ACO2, SUCLG1, FTCD, IDH3A, SDHA, COQ4, CTH, PANK3, HNF4A, PANK1, QPRT*
Fatty acid catabolic process	4.12E-06	*ACOX2, HACL1, ACOX1, CPT2, ACADM, ACADS, ETFDH, BDH2, DECR1, HADHA, ACAA1, HADHB*
Cholesterol efflux	5.75E-06	*APOA4, ABCG8, SOAT2, ABCG5, APOA1, APOC3, APOC2, SCARB1, APOM*
Transmembrane transport	6.80E-06	*SLC5A4, SLC22A18, CPT2, TRPV1, SLC5A1, MFSD2A, AQP7, AQP3, SLC25A20, SLC2A7, SLC23A1, APOA1, SLC2A5, SLC23A3, SLC2A2, SLC22A4, TRPV6, SLC22A5, FLVCR2, FLVCR1, TRPM6, SLC25A5, ABCC13, CNGA1, SLC26A3, SLC26A8, SLC25A34, CATSPER2, CATSPER3, SLC37A2, KCNH6, SLC30A10, SLC2A12, SLC39A14, SLC37A4, RHBG, KCNA3, SLC19A1, SLC47A2, MIP, SCARB1, SLC39A5, HCN4, SLC39A4, SLC2A9, ATP1A4, ABCB1, AQP10, SLC17A4, SLC13A2, ABCC2, SLC46A3, SCARA5, SLC25A15, ABCC8, PDZK1, SLC46A1, SLC5A11, SLC25A18, ABCC6, SLC5A12*
Carbohydrate transport	8.73E-06	*SLC2A12, SLC2A9, SLC5A4, SLC5A1, SLC37A4, AQP7, SLC2A7, G6PC, SLC2A5, AQP11, SLC2A2, SLC37A2, PLA2G1B, SCARB1, SLC5A11*
Response to nutrient levels	1.20E-05	*PPARA, CCK, HSD17B2, ALDOB, AQP3, VDR, PCSK1, UGT1A6, UGT1A8, CDKN2B, HMOX1, APOM, SOAT2, CYP1A1, ACADS, SI, CPS1, PPARGC1A, SLC6A19, SSTR2, G6PC, ABCG5, HMGCS2, GHRH, SSTR1, HSD11B2, GHRL, SST, LCT, KLF4*
Homeostatic process	1.23E-05	*XRCC5, JPH4, HNF1A, GRIK1, TRPV1, PDIA2, GNA11, EDN2, GRIK3, F2RL1, PDX1, APOA4, GSR, BAK1, APOB, APOA1, MALL, GRIN2B, AQP11, HMOX1, RGN, NPC1L1, CHRNA7, SLC22A5, CHRFAM7A, APOM, MB, FLVCR1, OTC, MLXIPL, GRIN2A, SLC9A3R1, PPARGC1A, CCR9, G6PC, HNF4A, GHRH, GHRL, MON1A, CALCR, XDH, PRKCZ, CCK, SLC37A4, DRD4, NR3C2, APOC2, SOX6, TRIM10, FKBP1B, VDR, APOC3, HAAO, SCARB1, SLC39A4, MT1H, GAL3ST1, GLRX, SOAT2, LIPA, TNFRSF13B, EPHX2, ATP1A4, AFG3L2, CPS1, CCL15, PCK1, MTTP, ABCG8, ABCG5, ADRA1B, NEUROD1, NR5A2, SCARA5, IL2*
Long-chain fatty acid transport	1.39E-05	*SLC25A20, ACE, CPT2, CD36, PLIN2, DRD4, PLA2G1B, FABP1, FABP2*

Digestive system process	1.65E-05	*ABCG8, VDR, SOAT2, SCT, MOGAT2, ABCG5, SLC5A1, NPC1L1, GHRL, FABP1, FABP2*
Vitamin metabolic process	2.47E-05	*ME1, CYP3A4, ALDH8A1, SHMT1, ACADM, CYP1A1, RBP2, SLC19A3, TPK1, CBR1, LRAT, ADH4, HAAO, SLC22A4, QPRT, RETSAT*
Glycerolipid metabolic process	2.49E-05	*PIP5K1B, APOC2, APOA4, APOB, PIP5KL1, APOA1, APOC3, AGPAT9, APOH, SLC22A4, PLA2G1B, CAT, NR1H3, GPD1, MOGAT2, PLD1, PNLIPRP2, CPS1, PCK1, MTTP, G6PC, DGAT1, DGAT2, SEMA6D, PLA2G2A*
Protein-lipid complex assembly	2.73E-05	*APOA4, SOAT2, APOB, APOA1, DGAT1, APOC3, APOM*
Plasma lipoprotein particle assembly	2.73E-05	*APOA4, SOAT2, APOB, APOA1, DGAT1, APOC3, APOM*
Response to carbohydrate stimulus	3.51E-05	*ME1, HNF1A, PFKFB2, SI, ALDOB, FKBP1B, ACVR1C, KHK, PCSK1, APOB, SARM1, HNF4A, NEUROD1, LCT*
Response to extracellular stimulus	3.59E-05	*PPARA, CCK, HSD17B2, ALDOB, AQP3, VDR, PCSK1, UGT1A6, UGT1A8, CDKN2B, HMOX1, APOM, SOAT2, CYP1A1, ACADS, SI, CPS1, PPARGC1A, SLC6A19, CDKN1A, SSTR2, G6PC, ABCG5, HMGCS2, GHRH, SSTR1, HSD11B2, GHRL, SST, LCT, KLF4*
Cellular amino acid derivative metabolic process	3.74E-05	*ENPP6, DRD4, GGT1, AGMAT, GCH1, APOA4, GSR, PADI6, APOA1, GPX3, SLC22A4, PLA2G1B, VNN1, SULT1A2, SULT1A4, DDC, ACADM, CYP1A1, MAOA, GRIN2A, CPS1, CKMT1A, CTH, SULT1B1, ABAT*
Lipid modification	3.87E-05	*ACOX2, HACL1, SOAT2, ACOX1, ACADM, CPT2, ACADS, DECR1, PPARGC1A, HADHA, HADHB, ETFDH, B4GALNT2, BDH2, ACAA1*
Cation transport	4.40E-05	*KCNJ16, SLC36A1, JPH4, SLC5A4, SLC22A18, CPT2, SLC20A2, TRPV1, SLC5A1, KCNK10, KCNJ13, SLC25A20, SLC23A1, GRIN2B, KCNK5, SLC22A4, CHRNA7, SLC22A5, TRPV6, CHRFAM7A, SLC4A4, TRPM6, SGK1, CLCA1, GRIN2A, CNGA1, CATSPER2, CATSPER3, SLC41A2, KCNH6, SLC30A10, SLC39A14, ORAI1, RHBG, CCL8, KCNA3, FKBP1B, KCNJ3, TMEM37, VDR, SLC4A7, SLC39A5, HCN4, SLC39A4, CAMK2A, SLC31A1, ATP1A4, ATP1A1, SLC10A2, KCNJ6, SLC17A4,*

		SLC13A2, KCTD16, ABCC8, SCARA5, PDZK1, SLC5A11, SLC5A12
Metal ion transport	4.81E-05	*KCNJ16, JPH4, ORAI1, SLC39A14, SLC5A4, TRPV1, SLC20A2, SLC5A1, KCNA3, CCL8, KCNJ3, FKBP1B, KCNJ13, KCNK10, VDR, TMEM37, SLC23A1, GRIN2B, KCNK5, SLC22A4, SLC4A7, CHRNA7, SLC22A5, SLC39A5, TRPV6, SLC4A4, SLC39A4, HCN4, CHRFAM7A, SLC31A1, CAMK2A, SGK1, TRPM6, CLCA1, GRIN2A, ATP1A4, ATP1A1, SLC10A2, CNGA1, KCNJ6, CATSPER2, SLC17A4, CATSPER3, KCNH6, SLC13A2, KCTD16, SLC30A10, SCARA5, ABCC8, SLC5A11, SLC5A12*
Peptide transport	5.36E-05	*EDN3, HNF1A, DGAT1, SLC15A1, TRPV1, CDH17, GHRH, FAM3B, NEUROD1, GHRL, PDX1, FKBP1B, SMPD3*
Gluconeogenesis	6.01E-05	*GPD1, G6PC, ALDOB, FBP1, PGAM2, GPT, PCK2, PPARGC1A, PCK1*
Alditol metabolic process	6.01E-05	*GALK1, GPD1, MOGAT2, DGAT2, DAK, GK, GDPD1, PCK1, GDPD2*
Amino acid transport	6.13E-05	*SLC36A1, CPT2, SLC16A10, SLC6A12, SLC7A9, SLC3A1, SLC19A1, SLC6A19, SLC7A7, SLC25A20, SLC6A5, SLC22A4, SLC22A5, SLC25A15, SLC1A1, PDZK1, SLC46A1*
Response to drug	6.58E-05	*CAV2, APOBEC1, SLC22A18, AQP7, HADHA, SLC47A2, PCSK1, UGT1A6, UGT1A8, PLIN2, SLC22A5, NAT8B, NAT8, CYP1A1, GRIN2A, ABCB1, ATP1A1, CPS1, ABCG2, CDKN1A, ABCG5, HMGCS2, ABAT, PEBP1, NEUROD1, CA4, HSD11B2, SST, PDZK1, LCT, ABCC6*
Polyol metabolic process	7.76E-05	*GALK1, GPD1, MOGAT2, DGAT2, DAK, PPIP5K1, GK, ITPKA, GDPD1, PCK1, GDPD2*
Xenobiotic metabolic process	9.23E-05	*CYP3A4, FMO4, UGT1A6, NR1I2, UGT1A8, HNF4A, CYP1A1, UGT2B11, EPHX2*
Regulation of cellular ketone metabolic process	9.40E-05	*APOA4, SLC25A20, PPARA, CPT2, HNF4A, BHMT, APOC3, MLXIPL, APOC2, FABP1, STRADB, PPARGC1A, NR1H3*
Peptide secretion	9.73E-05	*EDN3, HNF1A, DGAT1, TRPV1, GHRH, FAM3B, NEUROD1, GHRL, PDX1, FKBP1B, SMPD3*
Hormone transport	9.91E-05	*SHBG, EDN3, HNF1A, DGAT1, TBX3, GHRH, FAM3B, NEUROD1, GHRL, PDX1, FKBP1B, SMPD3*
Regulation of fatty acid metabolic process	9.91E-05	*APOA4, SLC25A20, PPARA, CPT2, HNF4A, APOC3, MLXIPL, APOC2, FABP1, STRADB, PPARGC1A, NR1H3*

Monosaccharide metabolic process	1.07E-04	*PFKFB4, PFKFB2, ALDOC, SLC37A4, ALDOB, PGAM2, PDX1, GALK1, GALM, B4GALNT2, GPD1, HKDC1, PDK4, GALT, FBP1, RBKS, CHST5, PCK2, PPP1CC, CPS1, PPARGC1A, PCK1, KHK, G6PC, CHST6, PKLR, GHRL, GPT, XYLB*
Excretion	1.12E-04	*ABCG8, SLC26A3, ABCG5, SLC22A18, TRPV1, HMOX1, KCNK5, NR3C2, AQP7, AMN, GUCA2B, AQP3, SCTR*
Pyruvate metabolic process	1.21E-04	*GPD1, G6PC, PFKFB2, ALDOB, FBP1, PGAM2, GPT, PCK2, AGXT, PPARGC1A, PCK1*
Positive regulation of fatty acid metabolic process	1.31E-04	*APOA4, PPARA, HNF4A, MLXIPL, APOC2, FABP1, PPARGC1A, NR1H3*
Regulation of secretion	1.33E-04	*EDN3, HNF1A, GRIK1, PFKFB2, EDN2, DRD4, KRT20, ADA, ACVR1C, PCSK1, APOA1, GRIN2B, HMOX1, PLA2G1B, TRPV6, RAB26, CASP1, NRG1, CAMK2A, SCAMP5, SIRT4, GCG, GHRH, SCIN, NEUROD1, GHRL, IL2*
Lipid digestion	1.35E-04	*APOA4, ABCG8, SOAT2, ABCG5, PLA2G1B, NPC1L1*
Hormone metabolic process	1.38E-04	*ALDH8A1, HSD17B11, CYP1A1, DDO, PCSK2, PCSK1, UGT1A6, ACE, APOA1, UGT1A8, LRAT, SULT1B1, ADH4, UGT2B11, HSD11B2, SCARB1, SULT1E1, PCSK5, RETSAT*
Nitrogen compound biosynthetic process	1.79E-04	*MOCOS, ME1, ALAD, SEPHS2, HNF1A, NAGS, ATP10A, AGMAT, AGXT, ADA, GCH1, TPK1, PADI6, ARG2, ENTPD8, HAAO, SULT1A2, GUCA2B, GCHFR, MOCS1, DDC, SHMT1, OTC, ATP1A4, ATP1A1, CPS1, GUCY2C, AMPD1, ADI1, CTH, ADCY9, BHMT, PRODH2, QPRT, DPYD, SLC25A15, PRODH*
Drug metabolic process	1.93E-04	*CYP3A4, CBR1, CYP1A1, CYP2C19, CYP2C9, CYP2D6, EPHX2*
Reverse cholesterol transport	1.93E-04	*APOA4, APOA1, HNF1A, APOC3, APOC2, SCARB1, APOM*
Hormone secretion	2.24E-04	*EDN3, HNF1A, DGAT1, TBX3, GHRH, FAM3B, NEUROD1, GHRL, PDX1, FKBP1B, SMPD3*
Regulation of cholesterol storage	2.36E-04	*PPARA, APOB, MSR1, CD36, SCARB1, NR1H3*
Response to xenobiotic stimulus	2.50E-04	*CYP3A4, FMO4, UGT1A6, NR1I2, UGT1A8, HNF4A, CYP1A1, UGT2B11, EPHX2*

Glycerol metabolic process	2.50E-04	*GPD1, MOGAT2, DGAT2, DAK, GK, GDPD1, PCK1, GDPD2*
Endocrine process	2.50E-04	*EDN3, ACE, TBX3, EDN2, ACE2, HSD11B2, GHRL, PCSK5*
Endocrine pancreas development	2.84E-04	*INSM1, HNF1A, ONECUT2, NEUROD1, IL6R, PDX1, NKX2-2*
Hexose biosynthetic process	3.20E-04	*GPD1, G6PC, ALDOB, FBP1, PGAM2, GPT, PCK2, PPARGC1A, PCK1*
Vitamin transport	3.35E-04	*SLC25A20, SLC23A1, CPT2, SLC22A4, SLC22A5, SLC19A1, PDZK1, SLC46A1*
Peptide hormone secretion	3.40E-04	*EDN3, HNF1A, DGAT1, GHRH, FAM3B, NEUROD1, GHRL, PDX1, FKBP1B, SMPD3*
Betaine transport	3.57E-04	*SLC25A20, CPT2, SLC22A4, SLC22A5, PDZK1*
Carnitine transport	3.57E-04	*SLC25A20, CPT2, SLC22A4, SLC22A5, PDZK1*
Amine transport	5.13E-04	*SLC36A1, CPT2, SLC16A10, SLC6A12, SLC6A4, SLC7A9, SLC3A1, SLC19A1, SLC6A19, SLC7A7, SLC25A20, SLC6A5, SLC22A4, SLC22A5, SLC25A15, PDZK1, SLC1A1, SLC46A1*
Cellular hormone metabolic process	5.57E-04	*HSD17B11, ALDH8A1, UGT1A6, LRAT, APOA1, UGT1A8, CYP1A1, ADH4, UGT2B11, HSD11B2, SCARB1, SULT1E1, RETSAT*
Response to glucocorticoid stimulus	5.64E-04	*CALCR, ACADS, SI, ALDOB, IL6R, CPS1, AGXT, TAT, PPARGC1B, PCSK1, UGT1A6, CDKN1A, UGT1A8, HSD11B2, PEBP1*
Regulation of system process	6.34E-04	*EDN3, PRKCZ, CCK, CYP2J2, GRIK1, GRIK3, EDN2, DRD4, FKBP1B, ADA, SYP, APOA4, ACE, APOA1, GRIN2B, HMOX1, ARG2, SLC22A5, LGI1, HCN4, CAMK2A, EPHX2, GRIN2A, MYH7, ATP1A1, CPS1, ABCG8, SSTR2, ABCG5, ADRA1B, ACE2, PEBP1, GHRL, IL2*
Unsaturated fatty acid metabolic process	6.51E-04	*AKR1C3, CYP4F8, ACOX1, PTGR1, CYP2J2, EDN2, EPHX2, PLA2G1B, CYP4F3, CYP4F2, HPGD*
Cofactor transport	7.64E-04	*SLC25A20, CPT2, SLC22A4, SLC22A5, SLC19A1, PDZK1, SLC46A1*
Macromolecular complex remodeling	7.64E-04	*APOA4, APOB, APOC3, PLA2G2A, APOC2, SCARB1, APOM*

Plasma lipoprotein particle remodeling	7.64E-04	*APOA4, APOB, APOC3, PLA2G2A, APOC2, SCARB1, APOM*
Regulation of cholesterol transport	7.64E-04	*APOA4, ABCG8, ABCG5, APOA1, APOC3, APOC2, NR1H3*
Regulation of sterol transport	7.64E-04	*APOA4, ABCG8, ABCG5, APOA1, APOC3, APOC2, NR1H3*
Protein-lipid complex remodeling	7.64E-04	*APOA4, APOB, APOC3, PLA2G2A, APOC2, SCARB1, APOM*
Regulation of lipid metabolic process	8.14E-04	*PPARA, CPT2, MLXIPL, APOC2, ATP1A1, STRADB, PPARGC1A, SEC14L2, APOA4, SLC25A20, APOB, APOA1, HNF4A, APOC3, FABP1, APOM, NR1H3*
Amine biosynthetic process	8.17E-04	*ADI1, DDC, SHMT1, SEPHS2, CTH, NAGS, OTC, BHMT, PRODH2, SULT1A2, AGMAT, AGXT, PRODH, GCH1*
Hexose metabolic process	8.84E-04	*GPD1, PFKFB4, PFKFB2, ALDOC, HKDC1, SLC37A4, PDK4, ALDOB, GALT, FBP1, PGAM2, PDX1, PPP1CC, PCK2, CPS1, PPARGC1A, PCK1, KHK, GALK1, GALM, G6PC, PKLR, GHRL, GPT*
Negative regulation of transport	9.10E-04	*EDN3, CRYAA, SLC15A1, EDN2, DRD4, SIRT4, APOC2, ADA, ACVR1C, ABCG8, ABCG5, APOA1, HNF4A, HMOX1, APOC3, GHRL, NRG1, NR1H3, SCAMP5*
Response to inorganic substance	9.57E-04	*XDH, APOBEC1, CYP1A1, ALDOB, PGAM2, MYH7, TAT, AQP3, ADA, APOA4, KHK, PCSK1, APOB, CDKN1A, HMOX1, GPX3, CYBRD1, HAAO, PEBP1, ABAT, CAT, CA2, MT1H, LCT, MB*
Monosaccharide biosynthetic process	1.15E-03	*GPD1, G6PC, ALDOB, FBP1, PGAM2, GPT, PCK2, PPARGC1A, PCK1*
Triglyceride biosynthetic process	1.17E-03	*MOGAT2, DGAT1, DGAT2, AGPAT9, PCK1*
Positive regulation of lipid metabolic process	1.22E-03	*APOA4, PPARA, APOA1, HNF4A, MLXIPL, APOC2, FABP1, PPARGC1A, SEC14L2, NR1H3*

Regulation of fatty acid biosynthetic process	1.26E-03	*APOA4, HNF4A, APOC3, MLXIPL, APOC2, NR1H3*
Response to corticosteroid stimulus	1.30E-03	*CALCR, ACADS, SI, ALDOB, IL6R, CPS1, AGXT, TAT, PPARGC1B, PCSK1, UGT1A6, CDKN1A, UGT1A8, HSD11B2, PEBP1*
Lipid biosynthetic process	1.35E-03	*ALDH8A1, HSD17B11, HNF1A, HSD17B2, EDN2, ACSS2, PDSS1, PECR, APOA1, B3GNT5, AGPAT9, PLA2G1B, PIWIL2, NPC1L1, HSD17B3, SCARB1, ELOVL7, PCYT2, GAL3ST1, ACAA2, MOGAT2, PLD1, CYP1A1, CDS1, LPCAT3, FADS6, PCK1, DGAT1, HMGCS2, ST8SIA6, DGAT2, SEMA6D, HSD11B2, SCP2*
Cellular amide metabolic process	1.39E-03	*ME1, GPD1, NAGS, ARG2, OTC, ALDOB, HAAO, QPRT, CPS1, SLC25A15, IDH3A*
Icosanoid metabolic process	1.43E-03	*AKR1C3, CYP4F8, ACOX1, PTGR1, CYP2J2, EDN2, PLA2G1B, CYP4F3, CYP4F2, HPGD*
Response to monosaccharide stimulus	1.43E-03	*KHK, PCSK1, SARM1, HNF1A, HNF4A, PFKFB2, SI, NEUROD1, FKBP1B, ACVR1C*
Response to hexose stimulus	1.43E-03	*KHK, PCSK1, SARM1, HNF1A, HNF4A, PFKFB2, SI, NEUROD1, FKBP1B, ACVR1C*
Response to organic substance	1.48E-03	*PPARA, HNF1A, APOBEC1, TLR3, FOXO4, AGXT, ACVR1C, APOB, CDKN2B, GRIN2B, HMOX1, PLA2G1B, CHRNA7, CREB3L3, CASP1, CHRFAM7A, APOM, MB, PLD1, CYP1A1, ACADS, FBP1, GRIN2A, IL6R, TAT, PPARGC1B, HNF4A, ERN1, GHRL, HSD11B2, PEBP1, ABAT, CA4, CA2, LCT, ME1, CALCR, CAV2, ALDOC, PFKFB2, DRD4, ALDOB, ADH6, LIN28A, FKBP1B, GCH1, UGT1A6, PCSK1, UGT1A8, PLIN2, SCARB1, KAT2B, SI, CPS1, GNAT3, PCK1, GCG, KHK, CDKN1A, SARM1, ADCY9, HMGCS2, ADRA1B, NEUROD1, SST*
Biogenic amine metabolic process	1.53E-03	*ENPP6, DDC, ACADM, MAOA, DRD4, GRIN2A, AGMAT, GCH1, APOA4, APOA1, SULT1B1, PLA2G1B, SLC22A4, SULT1A2, SULT1A4*
Sodium ion transport	1.55E-03	*SGK1, SLC5A4, SLC20A2, SLC5A1, ATP1A4, ATP1A1, SLC10A2, SLC23A1, CATSPER3, SLC17A4, SLC22A4, SLC13A2, SLC4A7, SLC22A5, HCN4, SLC4A4, SLC5A11, SLC5A12*

Glucose metabolic process	1.57E-03	*GPD1, PFKFB2, ALDOC, HKDC1, SLC37A4, PDK4, ALDOB, GALT, FBP1, PGAM2, PDX1, PPP1CC, CPS1, PCK2, PPARGC1A, PCK1, G6PC, PKLR, GHRL, GPT*
Response to endogenous stimulus	1.58E-03	*CALCR, ME1, CAV2, PPARA, APOBEC1, ALDOC, ALDOB, DRD4, FOXO4, AGXT, ACVR1C, UGT1A6, PCSK1, UGT1A8, HMOX1, PLA2G1B, MB, PLD1, KAT2B, ACADS, SI, GRIN2A, FBP1, IL6R, CPS1, TAT, PPARGC1B, PCK1, KHK, GCG, CDKN1A, ADCY9, HMGCS2, ADRA1B, PEBP1, GHRL, HSD11B2, CA4, CA2, SST, LCT*
Organic cation transport	1.71E-03	*SLC25A20, CPT2, SLC22A18, SLC22A4, RHBG, SLC22A5, PDZK1*
Positive regulation of cholesterol storage	1.84E-03	*APOB, MSR1, CD36, SCARB1*
Urea metabolic process	1.86E-03	*NAGS, ARG2, OTC, CPS1, SLC25A15*
Urea cycle	1.86E-03	*NAGS, ARG2, OTC, CPS1, SLC25A15*
Lipoprotein transport	1.86E-03	*APOB, MSR1, APOBEC1, CD36, MTTP*
Regulation of blood pressure	2.05E-03	*EDN3, PPARA, EDN2, EPHX2, ATP1A1, GCH1, ACE, CHGA, HMOX1, ADRA1B, ACE2, ABAT, HSD11B2, GUCA2B, PCSK5*
Coenzyme biosynthetic process	2.13E-03	*MOCOS, ME1, TPK1, COQ4, PANK3, PANK1, HAAO, GGT1, QPRT, PDSS1, MOCS1, GCH1*
Sterol metabolic process	2.26E-03	*ACAA2, SOAT2, HNF1A, LIPA, NR0B2, APOA4, APOB, APOA1, CYP27A1, HMGCS2, CYP7A1, APOC3, NPC1L1, SCARB1, CAT*
Fructose metabolic process	2.35E-03	*KHK, PFKFB4, PFKFB2, ALDOC, ALDOB, FBP1*
Regulation of lipoprotein lipase activity	2.35E-03	*APOA4, APOC3, APOH, APOC2, FKBP1B, NR1H3*
Cellular amino acid biosynthetic process	2.60E-03	*ADI1, SHMT1, CTH, SEPHS2, NAGS, OTC, BHMT, PRODH2, AGXT, PRODH*
Fat-soluble vitamin metabolic process	2.61E-03	*CYP3A4, ALDH8A1, CBR1, LRAT, CYP1A1, ADH4, RBP2, RETSAT*

Cholesterol metabolic process	2.69E-03	*ACAA2, SOAT2, HNF1A, NR0B2, APOA4, APOB, APOA1, CYP27A1, HMGCS2, CYP7A1, APOC3, NPC1L1, SCARB1, CAT*
Regulation of digestive system process	2.79E-03	*APOA4, ABCG8, ABCG5, APOA1, SLC22A5*
Amide biosynthetic process	2.79E-03	*NAGS, ARG2, OTC, CPS1, SLC25A15*
Positive regulation of fatty acid biosynthetic process	2.79E-03	*APOA4, HNF4A, MLXIPL, APOC2, NR1H3*
Response to hormone stimulus	3.16E-03	*CALCR, ME1, CAV2, PPARA, APOBEC1, ALDOB, FOXO4, AGXT, ACVR1C, UGT1A6, PCSK1, UGT1A8, HMOX1, PLA2G1B, MB, PLD1, KAT2B, ACADS, SI, FBP1, IL6R, CPS1, TAT, PPARGC1B, PCK1, KHK, GCG, CDKN1A, ADCY9, HMGCS2, PEBP1, GHRL, HSD11B2, CA4, CA2, SST, LCT*
Response to nutrient	3.44E-03	*SOAT2, CYP1A1, HSD17B2, SI, SLC6A19, AQP3, VDR, UGT1A6, SSTR2, ABCG5, UGT1A8, CDKN2B, HMGCS2, SSTR1, HMOX1, SST, APOM, KLF4, LCT*
Negative regulation of multicellular organismal process	3.49E-03	*F11, GRIK1, GRIK3, APOC2, ATP1A1, IL6R, CXADR, FKBP1B, ADA, ABCG8, ABCG5, APOA1, HMOX1, ARG2, KLKB1, APOC3, APOH, GHRL, CHRNA7, CHRFAM7A, IL2*
High-density lipoprotein particle clearance	3.52E-03	*APOA1, APOC2, SCARB1, APOM*
Regulation of intestinal cholesterol absorption	3.52E-03	*APOA4, ABCG8, ABCG5, APOA1*
Very-low-density lipoprotein particle assembly	3.52E-03	*SOAT2, APOB, DGAT1, APOC3*
Response to metal ion	3.61E-03	*XDH, APOBEC1, CYP1A1, ALDOB, PGAM2, TAT, AQP3, KHK, PCSK1, APOB, CYBRD1, HAAO, ABAT, PEBP1, CA2, MT1H, LCT*

Alcohol biosynthetic process	3.66E-03	*GPD1, G6PC, ALDOB, FBP1, PGAM2, GPT, PCK2, PPARGC1A, PCK1*
Pancreas development	3.71E-03	*INSM1, PCSK1, HNF1A, ONECUT2, NEUROD1, IL6R, PDX1, NKX2-2*
Phenol metabolic process	3.71E-03	*DDC, SULT1B1, MAOA, DRD4, GRIN2A, SULT1A2, SULT1A4, GCH1*
Cellular carbohydrate catabolic process	3.89E-03	*GPD1, ALDOC, HKDC1, PFKFB2, ALDOB, PGAM2, CPS1, GBA3, GBA2, G6PC, PKLR, MGAM, TREH*
Quaternary ammonium group transport	3.99E-03	*SLC25A20, CPT2, SLC22A4, SLC22A5, PDZK1*
Neutral lipid biosynthetic process	3.99E-03	*MOGAT2, DGAT1, DGAT2, AGPAT9, PCK1*
Acylglycerol biosynthetic process	3.99E-03	*MOGAT2, DGAT1, DGAT2, AGPAT9, PCK1*
Bile acid metabolic process	4.01E-03	*ACOX2, GBA2, HNF1A, SULT2A1, PIWIL2, NR5A2*
Regulation of systemic arterial blood pressure mediated by a chemical signal	4.10E-03	*EDN3, ACE, EDN2, ADRA1B, ACE2, HSD11B2, PCSK5*
Regulation of lipid biosynthetic process	4.23E-03	*APOA4, APOB, HNF4A, APOC3, MLXIPL, APOC2, ATP1A1, SEC14L2, NR1H3*
Response to glucose stimulus	4.23E-03	*KHK, PCSK1, SARM1, HNF1A, HNF4A, PFKFB2, NEUROD1, FKBP1B, ACVR1C*
Cofactor biosynthetic process	4.29E-03	*ME1, MOCOS, ALAD, HNF1A, GGT1, PDSS1, GCH1, COQ4, TPK1, PANK3, PANK1, HAAO, QPRT, MOCS1*
Carboxylic acid biosynthetic process	4.29E-03	*SHMT1, SEPHS2, PLD1, HNF1A, NAGS, OTC, EDN2, AGXT, FADS6, ADI1, PECR, CTH, BHMT, PLA2G1B, HAAO, PIWIL2, PRODH2, ELOVL7, PRODH*

Organic acid biosynthetic process	4.29E-03	*SHMT1, SEPHS2, PLD1, HNF1A, NAGS, OTC, EDN2, AGXT, FADS6, ADI1, PECR, CTH, BHMT, PLA2G1B, HAAO, PIWIL2, PRODH2, ELOVL7, PRODH*
Negative regulation of secretion	4.40E-03	*EDN3, APOA1, HMOX1, EDN2, DRD4, SIRT4, GHRL, NRG1, ADA, ACVR1C*
Cellular aldehyde metabolic process	4.97E-03	*ALDH1A1, ALDH8A1, AKR1B15, ADH4, AKR1B10, AKR7A2, AKR7A3, AGXT*
Regulation of leukocyte migration	5.09E-03	*EDN3, HMOX1, EDN2, F2RL1, IL6R, ADA*
Regulation of systemic arterial blood pressure by hormone	5.09E-03	*EDN3, ACE, EDN2, ACE2, HSD11B2, PCSK5*
Quinone cofactor metabolic process	5.50E-03	*COQ4, CBR1, ADH4, PDSS1, CRYZL1*
Glycerol ether biosynthetic process	5.50E-03	*MOGAT2, DGAT1, DGAT2, AGPAT9, PCK1*
Regulation of cellular localization	5.83E-03	*EDN3, HNF1A, CRYAA, PFKFB2, EDN2, DRD4, SIRT4, KRT20, FKBP1B, ACVR1C, GCG, PCSK1, APOA1, HNF4A, GRIN2B, GHRH, HMOX1, PLA2G1B, NEUROD1, GHRL, TRPV6, RAB26, CASP1, CAMK2A, IL2, SCAMP5*
Negative regulation of sterol transport	5.90E-03	*ABCG8, ABCG5, APOC3, APOC2*
Negative regulation of cholesterol transport	5.90E-03	*ABCG8, ABCG5, APOC3, APOC2*
Positive regulation of lipoprotein lipase activity	5.90E-03	*APOA4, APOH, APOC2, NR1H3*
Glucose transport	5.96E-03	*SLC2A9, G6PC, SLC2A5, SLC5A1, SLC2A2, SLC37A4, PLA2G1B*
Regulation of lipid storage	6.37E-03	*PPARA, APOB, MSR1, CD36, SCARB1, NR1H3*

Positive regulation of lipid biosynthetic process	6.37E-03	*APOA4, HNF4A, MLXIPL, APOC2, SEC14L2, NR1H3*
Endocrine system development	6.70E-03	*INSM1, PCSK1, APOA1, HNF1A, GHRH, ONECUT2, NEUROD1, IL6R, PDX1, MDK, NKX2-2*
Lipoprotein metabolic process	6.83E-03	*APOA4, PPARA, APOB, APOBEC1, CD36, APOA1, SEMA6D, APOC3, NPC1L1, APOM, APOL5, MTTP*
Hexose transport	7.09E-03	*SLC2A9, G6PC, SLC2A5, SLC5A1, SLC2A2, SLC37A4, PLA2G1B*
Regulation of lipid transport	7.09E-03	*APOA4, ABCG8, ABCG5, APOA1, APOC3, APOC2, NR1H3*
Carbohydrate homeostasis	7.20E-03	*G6PC, HNF1A, SLC37A4, MLXIPL, ADRA1B, NEUROD1, PDX1, PPARGC1A, PCK1*
Glucose homeostasis	7.20E-03	*G6PC, HNF1A, SLC37A4, MLXIPL, ADRA1B, NEUROD1, PDX1, PPARGC1A, PCK1*
Positive regulation of heart contraction	7.34E-03	*EDN3, EDN2, ADRA1B, ATP1A1, ADA*
Sulfur metabolic process	7.36E-03	*SEPHS2, GGT1, SLC19A3, CHST5, ADI1, TPK1, SULT4A1, GSR, MSRA, CTH, CHST6, BHMT, GPX3, CHST13, SEPP1*
Regulation of heart rate	7.85E-03	*EDN3, EDN2, ADRA1B, MYH7, FKBP1B, ADA*
Water-soluble vitamin metabolic process	7.97E-03	*ME1, TPK1, SHMT1, ACADM, SLC22A4, HAAO, SLC19A3, QPRT*
Monosaccharide transport	8.36E-03	*SLC2A9, G6PC, SLC2A5, SLC5A1, SLC2A2, SLC37A4, PLA2G1B*
Response to peptide hormone stimulus	8.93E-03	*PPARA, CAV2, PLD1, KAT2B, APOBEC1, SI, ALDOB, FBP1, IL6R, FOXO4, CPS1, PCK1, ACVR1C, KHK, PCSK1, HMGCS2, PLA2G1B, HSD11B2*
Intestinal cholesterol absorption	9.03E-03	*ABCG8, SOAT2, ABCG5, NPC1L1*
Response to steroid hormone stimulus	9.07E-03	*CALCR, CAV2, ACADS, SI, ALDOB, IL6R, CPS1, AGXT, TAT, PPARGC1B, PCSK1, UGT1A6, CDKN1A, UGT1A8, HMOX1, PEBP1, CA4, HSD11B2, GHRL, CA2, SST, LCT*

Positive regulation of multicellular organismal process	9.13E-03	*MAVS, PRKCZ, EDN3, GRIK1, KL, TRPV1, EDN2, EPHX2, TLR3, ATP1A1, IL6R, CPS1, ADA, CARD11, GHRH, HMOX1, GATA4, ADRA1B, APOH, GHRL, SLC22A5, CA2, LGI1, NRG1, CASP1*
Vitamin A metabolic process	9.55E-03	*ALDH8A1, LRAT, CYP1A1, ADH4, RBP2, RETSAT*
Negative regulation of lipid transport	9.55E-03	*ABCG8, ABCG5, APOC3, APOC2, NR1H3*
Carbohydrate biosynthetic process	9.77E-03	*B3GNT8, GPD1, G6PC, GCNT2, ST8SIA6, CHST6, ALDOB, FBP1, CHST13, PGAM2, GPT, PCK2, PPARGC1A, PCK1*
Stem cell differentiation	9.79E-03	*XRCC5, ACE, CDX2, PLA2G2A, PIWIL2, LIN28A, KLF4*
Glycoside catabolic process	9.92E-03	*GBA3, GBA2, TREH*
Generation of a signal involved in cell-cell signaling	1.07E-02	*EDN3, HNF1A, DGAT1, TBX3, BAIAP3, GHRH, FAM3B, NEUROD1, GHRL, PDX1, FKBP1B, SMPD3*
Steroid biosynthetic process	1.07E-02	*HSD17B11, ACAA2, APOA1, HNF1A, HSD17B2, HMGCS2, PIWIL2, HSD11B2, NPC1L1, SCARB1, HSD17B3, SCP2*
Phospholipid transport	1.14E-02	*APOA4, ABCG8, APOA1, APOC3, ATP10A, APOC2, ABCA4*
Peptide metabolic process	1.15E-02	*GSR, PCSK1, CTH, ACE, ADAMTS13, GPX3, MME, GGT1, PCSK5*
Cell-cell signaling	1.16E-02	*EDN3, HNF1A, GRIK1, GABRB2, FAM3B, SLC6A4, ENPEP, PDX1, VIPR1, WNT3, GRIN2B, GATA4, HTR1D, SLC1A1, GABRG1, BAIAP3, GRIN2A, IL26, NRXN1, SSTR2, DGAT1, GHRH, SSTR1, ABAT, PEBP1, GHRL, CAV2, DRD4, MME, CCL8, AKAP9, FKBP1B, PCSK1, PCSK5, GCHFR, MLN, BMP3, TRHDE, TBX3, CPNE6, MAOA, CCL15, CXCL14, SLC6A5, ADRA1B, MTNR1B, NEUROD1, SST, SMPD3, IL2*
Response to ethanol	1.19E-02	*UGT1A6, APOBEC1, UGT1A8, GRIN2B, DRD4, GRIN2A, ADH6, ABAT, PEBP1, IL6R, LCT*
Water transport	1.22E-02	*MIP, AQP11, AQP7, PDZD3, AQP3*

Monovalent inorganic cation transport	1.23E-02	*KCNJ16, SLC36A1, SLC5A4, SLC20A2, SLC5A1, KCNA3, KCNJ3, KCNK10, KCNJ13, SLC23A1, KCNK5, SLC22A4, SLC4A7, SLC22A5, HCN4, SLC4A4, SGK1, ATP1A4, ATP1A1, SLC10A2, CNGA1, KCNJ6, CATSPER3, SLC17A4, KCNH6, SLC13A2, KCTD16, ABCC8, SLC5A11, SLC5A12*
Positive regulation of lipid storage	1.30E-02	*APOB, MSR1, CD36, SCARB1*
Response to food	1.30E-02	*G6PC, CYP1A1, GHRH, HSD11B2*
Positive regulation of behavior	1.32E-02	*EDN3, GHRH, EDN2, F2RL1, GHRL, IL6R, SCG2*
Diol metabolic process	1.32E-02	*DDC, MAOA, DRD4, GRIN2A, SULT1A2, SULT1A4, GCH1*
Catechol metabolic process	1.32E-02	*DDC, MAOA, DRD4, GRIN2A, SULT1A2, SULT1A4, GCH1*
Catecholamine metabolic process	1.32E-02	*DDC, MAOA, DRD4, GRIN2A, SULT1A2, SULT1A4, GCH1*
Dicarboxylic acid metabolic process	1.32E-02	*SDHA, ME1, SUCLG1, HAAO, QPRT, IDH3A, PCK1*
Isoprenoid metabolic process	1.34E-02	*ALDH8A1, LRAT, CYP1A1, HMGCS2, ADH4, RBP2, PDSS1, RETSAT*
Regulation of foam cell differentiation	1.37E-02	*PPARA, APOB, MSR1, CD36, PLA2G2A, NR1H3*
Protein oligomerization	1.37E-02	*SHMT1, PRKCZ, CAV2, CRYAA, OTC, ALDOC, DECR1, PDSS1, GCH1, CTH, P2RX6, AQP11, GPX3, PEBP1, QPRT, CAT, SCARA5, SLC1A1, GCHFR*
Regulation of heart contraction	1.42E-02	*EDN3, CYP2J2, EDN2, ADRA1B, PEBP1, MYH7, ATP1A1, HCN4, FKBP1B, ADA, IL2*
Heterocycle catabolic process	1.42E-02	*ALDH6A1, UGT1A6, UGT1A8, CYP1A1, HMOX1, PRODH2, MYH7, DPYD, ADA, PRODH, GCH1, ABCC6*
Regulation of hormone secretion	1.45E-02	*GCG, EDN3, HNF1A, GHRH, PFKFB2, EDN2, SIRT4, NEUROD1, GHRL, ACVR1C*
Regulation of systemic arterial blood pressure	1.51E-02	*EDN3, ACE, EDN2, ADRA1B, ACE2, HSD11B2, PCSK5*

Fluid transport	1.52E-02	*MIP, AQP11, AQP7, PDZD3, AQP3*
Response to zinc ion	1.52E-02	*KHK, APOBEC1, ALDOB, HAAO, CA2*
Cellular chemical homeostasis	1.56E-02	*CALCR, PRKCZ, JPH4, CCK, GRIK1, GRIK3, GNA11, EDN2, F2RL1, DRD4, NR3C2, FKBP1B, VDR, BAK1, GRIN2B, AQP11, RGN, CHRNA7, CHRFAM7A, SLC39A4, MT1H, GAL3ST1, GRIN2A, EPHX2, ATP1A4, SLC9A3R1, AFG3L2, CCL15, PPARGC1A, CCR9, GHRH, GHRL, SCARA5, MON1A, IL2*
Nitrogen compound catabolic process	1.59E-02	*ALDH6A1, UGT1A6, UGT1A8, HMOX1, ENPP3, MYH7, DPYD, ADA, MPST, GCH1, ABCC6*
Ion homeostasis	1.61E-02	*CALCR, PRKCZ, JPH4, CCK, GRIK1, GRIK3, GNA11, EDN2, DRD4, F2RL1, NR3C2, FKBP1B, VDR, BAK1, GRIN2B, AQP11, HMOX1, RGN, CHRNA7, CHRFAM7A, SLC39A4, MT1H, GAL3ST1, OTC, GRIN2A, EPHX2, ATP1A4, CPS1, SLC9A3R1, AFG3L2, CCL15, CCR9, GHRH, GHRL, SCARA5, MON1A, IL2*
Secondary metabolic process	1.68E-02	*ME1, ALDH8A1, GPD1, LRAT, CYP1A1, ADH4, ALDOB, HAAO, QPRT, IDH3A, RETSAT*
Regulation of peptide secretion	1.69E-02	*GCG, HNF1A, GHRH, PFKFB2, SIRT4, NEUROD1, GHRL, ACVR1C*
Regulation of behavior	1.69E-02	*EDN3, GHRH, EDN2, F2RL1, GHRL, IL6R, ADA, SCG2*
Positive regulation of leukocyte chemotaxis	1.77E-02	*EDN3, EDN2, F2RL1, IL6R*
Positive regulation of heart rate	1.77E-02	*EDN3, EDN2, ADRA1B, ADA*
Phospholipid efflux	1.77E-02	*APOA4, APOA1, APOC3, APOC2*
Acid secretion	1.86E-02	*ACE, DRD4, PLA2G1B, GHRL, AGXT*
Insulin secretion	1.89E-02	*HNF1A, DGAT1, FAM3B, NEUROD1, PDX1, FKBP1B*
Cellular carbohydrate biosynthetic process	1.90E-02	*B3GNT8, GPD1, G6PC, ALDOB, FBP1, PGAM2, GPT, PCK2, PPARGC1A, PCK1*

Glycoside metabolic process	1.91E-02	*GBA3, GBA2, TREH*
Plasminogen activation	1.91E-02	*F11, KLKB1, APOH*
Secretion by cell	1.95E-02	*EDN3, HNF1A, TBX3, BAIAP3, FAM3B, CCL8, PDX1, FKBP1B, STXBP5L, TRIM36, DGAT1, GHRH, SCIN, GHRL, NEUROD1, DOC2B, PCSK5, SMPD3, MON1A, SCG2, SCAMP5*
Calcium ion transport	1.95E-02	*ORAI1, JPH4, TRPM6, CLCA1, TRPV1, GRIN2A, CCL8, FKBP1B, VDR, TMEM37, CATSPER2, GRIN2B, CATSPER3, CHRNA7, TRPV6, CHRFAM7A, CAMK2A*
Cellular ion homeostasis	2.10E-02	*CALCR, JPH4, PRKCZ, CCK, GRIK1, GRIK3, GNA11, EDN2, F2RL1, DRD4, NR3C2, FKBP1B, VDR, BAK1, GRIN2B, AQP11, RGN, CHRNA7, CHRFAM7A, SLC39A4, MT1H, GAL3ST1, GRIN2A, EPHX2, ATP1A4, SLC9A3R1, AFG3L2, CCL15, CCR9, GHRH, GHRL, SCARA5, MON1A, IL2*
Regulation of fatty acid oxidation	2.19E-02	*SLC25A20, PPARA, CPT2, FABP1, STRADB, PPARGC1A*
Vitamin biosynthetic process	2.19E-02	*ME1, TPK1, ALDH8A1, CYP1A1, HAAO, QPRT*
Prostaglandin metabolic process	2.25E-02	*AKR1C3, CYP4F8, ACOX1, EDN2, HPGD*
Prostanoid metabolic process	2.25E-02	*AKR1C3, CYP4F8, ACOX1, EDN2, HPGD*
Serine family amino acid biosynthetic process	2.33E-02	*SHMT1, CTH, SEPHS2, AGXT*
Sphingomyelin metabolic process	2.33E-02	*ENPP7, SMPDL3B, SMPDL3A, SMPD3*
Regulation of leukocyte chemotaxis	2.33E-02	*EDN3, EDN2, F2RL1, IL6R*
Protein tetramerization	2.49E-02	*SHMT1, CTH, ALDOC, GPX3, DECR1, CAT, PDSS1*

Response to organic cyclic substance	2.51E-02	*CYP1A1, ALDOC, DRD4, TAT, GNAT3, PCSK1, CDKN1A, PLIN2, CDKN2B, HMOX1, ADRA1B, PEBP1, ABAT, CHRNA7, CHRFAM7A*
Sulfur compound biosynthetic process	2.58E-02	*TPK1, ADI1, CTH, SEPHS2, CHST6, BHMT, CHST13, GGT1*
Carbohydrate catabolic process	2.60E-02	*GPD1, ALDOC, HKDC1, PFKFB2, ALDOB, PGAM2, CPS1, GBA3, GBA2, G6PC, PKLR, MGAM, TREH*
Positive regulation of secretion	2.60E-02	*EDN3, PCSK1, GRIN2B, GRIK1, GHRH, PFKFB2, EDN2, SCIN, PLA2G1B, GHRL, CASP1, IL2, SCAMP5*
Organophosphate metabolic process	2.61E-02	*GPD1, PLD1, ENPP7, GALT, PIP5K1B, CDS1, LPCAT3, APOA4, PLCB3, APOA1, PIP5KL1, SMPDL3B, SEMA6D, SMPDL3A, AGPAT9, PLA2G2A, PLA2G1B, GK, PCYT2, SMPD3*
Regulation of vasodilation	2.69E-02	*ACE, HMOX1, ACE2, EPHX2, CPS1*
Regulation of insulin secretion	2.79E-02	*GCG, HNF1A, PFKFB2, SIRT4, NEUROD1, GHRL, ACVR1C*
Di-, tri-valent inorganic cation transport	2.97E-02	*ORAI1, SLC39A14, JPH4, TRPM6, CLCA1, TRPV1, GRIN2A, CCL8, FKBP1B, TMEM37, VDR, CATSPER2, GRIN2B, CATSPER3, CHRNA7, TRPV6, CHRFAM7A, SCARA5, CAMK2A*
High-density lipoprotein particle remodeling	2.98E-02	*APOA4, APOC3, SCARB1, APOM*
Regulation of very-low-density lipoprotein particle remodeling	3.06E-02	*APOA1, APOC3, APOC2*
Sphingomyelin catabolic process	3.06E-02	*SMPDL3B, SMPDL3A, SMPD3*
Oxidoreduction coenzyme metabolic process	3.13E-02	*ME1, COQ4, GPD1, ALDOB, HAAO, QPRT, PDSS1, IDH3A*
Cofactor catabolic process	3.28E-02	*SDHA, UGT1A6, UGT1A8, ACO2, HMOX1, SUCLG1, IDH3A*

Response to starvation	3.46E-02	*UGT1A6, UGT1A8, ACADS, ALDOB, SI, CPS1, PPARGC1A, LCT*
Phospholipid catabolic process	3.70E-02	*PLCB3, PLD1, SMPDL3B, SMPDL3A, SMPD3*
Nitric oxide metabolic process	3.70E-02	*ARG2, CPS1, GCHFR, GCH1*
Positive regulation of foam cell differentiation	3.70E-02	*APOB, MSR1, CD36, PLA2G2A*
Positive regulation of lipid catabolic process	3.70E-02	*APOA4, PPARA, APOC2, FABP1*
Drug transport	3.70E-02	*SLC22A18, SLC22A5, PDZK1, SLC47A2*
Regulation of body fluid levels	3.74E-02	*F11, F10, ADAMTS13, F2RL1, MST1, F13B, SCT, CD36, HNF4A, KLKB1, PROZ, SLC22A4, APOH, HSD11B2, GUCA2B*
Response to oxygen levels	3.74E-02	*PPARA, CYP1A1, PDIA2, ALDOC, ADA, ACE, CDKN1A, HMOX1, ABAT, HSD11B2, CHRNA7, CHRFAM7A, CASP1, LCT, DPP4, MB*
Cellular homeostasis	3.77E-02	*CALCR, PRKCZ, JPH4, CCK, GRIK1, PDIA2, GRIK3, GNA11, EDN2, DRD4, F2RL1, NR3C2, FKBP1B, GSR, VDR, BAK1, GRIN2B, AQP11, HAAO, RGN, CHRNA7, CHRFAM7A, SLC39A4, MT1H, GAL3ST1, GLRX, GRIN2A, EPHX2, ATP1A4, SLC9A3R1, AFG3L2, CCL15, PPARGC1A, CCR9, GHRH, GHRL, SCARA5, MON1A, IL2*
Amine catabolic process	3.85E-02	*ALDH6A1, SHMT1, ASPA, MAOA, AMT, GLS, PRODH2, TAT, DDO, PRODH*
Diterpenoid metabolic process	4.28E-02	*ALDH8A1, LRAT, CYP1A1, ADH4, RETSAT*
Retinoid metabolic process	4.28E-02	*ALDH8A1, LRAT, CYP1A1, ADH4, RETSAT*
Nucleobase metabolic process	4.28E-02	*ALDH6A1, SHMT1, DPYD, ADA, AMPD1*
Sulfur amino acid metabolic process	4.28E-02	*ADI1, MSRA, CTH, SEPHS2, BHMT*
Regulation of postsynaptic	4.28E-02	*GRIN2B, GRIK1, DRD4, GRIN2A, GHRL*

membrane potential		
Cellular cation homeostasis	4.38E-02	*CALCR, JPH4, EDN2, F2RL1, DRD4, EPHX2, NR3C2, ATP1A4, CCL15, FKBP1B, CCR9, BAK1, VDR, AQP11, GHRH, RGN, GHRL, CHRNA7, CHRFAM7A, SLC39A4, MT1H, SCARA5, IL2, MON1A*
Regulation of triglyceride catabolic process	4.41E-02	*APOA4, APOC3, APOC2*
Negative regulation of lipoprotein metabolic process	4.41E-02	*APOA4, APOM, HHATL*
Cellular amino acid catabolic process	4.49E-02	*ALDH6A1, SHMT1, ASPA, AMT, GLS, PRODH2, TAT, DDO, PRODH*
Nitric oxide mediated signal transduction	4.51E-02	*CD36, NEUROD1, PDX1, MT1H*
Purine base metabolic process	4.51E-02	*SHMT1, DPYD, ADA, AMPD1*
Circulatory system process	4.64E-02	*EDN3, CAV2, PPARA, EDN2, EPHX2, ATP1A1, GCH1, ACE, CHGA, NTS, HMOX1, ADRA1B, ACE2, ABAT, HSD11B2, GUCA2B, HCN4, PCSK5*
Blood circulation	4.64E-02	*EDN3, CAV2, PPARA, EDN2, EPHX2, ATP1A1, GCH1, ACE, CHGA, NTS, HMOX1, ADRA1B, ACE2, ABAT, HSD11B2, GUCA2B, HCN4, PCSK5*
Negative regulation of phosphorylation	4.64E-02	*BAK1, PRKCZ, CDKN1A, HNF4A, CDKN2B, PEBP1, IL2*
Regulation of response to external stimulus	4.69E-02	*F11, EDN3, PPARA, CCK, EDN2, F2RL1, IL6R, ADA, KLKB1, ACE2, PLA2G2A, APOH, GHRL, CHRNA7, CHRFAM7A, IL2, SCG2*
Nucleoside triphosphate catabolic process	4.90E-02	*ENPP3, MYH7, ADA, GCH1, ABCC6*

Aspartate family amino acid metabolic process	4.90E-02	*ADI1, MSRA, ASPA, BHMT, DDO*
Brown fat cell differentiation	4.90E-02	*PEX11A, ALDH6A1, PRDM16, PPARGC1A, MB*
Visual perception	5.09E-02	*GJA10, CLRN1, CRYAA, KRT12, CYP4V2, CDS1, ABCA4, PHYH, CNGA1, RDH5, SFRP5, MIP, CRYGC, LRAT, NRL, CA4, USH1C, ACAD11, OAT, ABCC6*
Sensory perception of light stimulus	5.09E-02	*GJA10, CLRN1, CRYAA, KRT12, CYP4V2, CDS1, ABCA4, PHYH, CNGA1, RDH5, SFRP5, MIP, CRYGC, LRAT, NRL, CA4, USH1C, ACAD11, OAT, ABCC6*

Supplementary Table 4. Connectivity Map analysis of differentially expressed genes (DEGs) from ampullary cancer microarray. Chemicals with a negative correlation with DEGs of ampullary cancer are those with higher potential of cytotoxic ability.

Rank	CMap name	Dose	Cell	Score	Up	Down	Instance id
5489	fludrocortisone	9 µM	HL60	0	-0.19	-0.234	2368
5490	SB-202190	1 µM	PC3	0	-0.19	-0.14	7058
5491	tocainide	17 µM	MCF7	0	-0.19	-0.195	4838
5492	diclofenamide	13 µM	MCF7	0	-0.19	-0.225	5286
5493	tribenoside	8 µM	HL60	0	-0.19	-0.159	2946
5494	(-)-isoprenaline	16 µM	PC3	0	-0.19	-0.093	4495
5495	harman	18 µM	HL60	0	-0.191	-0.22	2150
5496	phentolamine	12 µM	MCF7	0	-0.191	-0.207	1138
5497	rosiglitazone	10 µM	MCF7	0	-0.191	-0.235	6950
5498	rilmenidine	8 µM	PC3	0	-0.191	-0.129	5107
5499	bacampicillin	8 µM	MCF7	0	-0.191	-0.238	3273
5500	hydroflumethiazide	12 µM	PC3	0	-0.191	-0.139	1809
5501	lobeline	11 µM	MCF7	0	-0.191	-0.161	6258
5502	megestrol	10 µM	HL60	0	-0.191	-0.206	3091
5503	iocetamic acid	7 µM	PC3	0	-0.191	-0.169	4600
5504	isoxicam	12 µM	PC3	0	-0.191	-0.147	7268
5505	haloperidol	10 µM	PC3	0	-0.191	-0.119	1244
5506	dihydroergocristine	6 µM	MCF7	0	-0.191	-0.195	7034
5507	AG-013608	10 µM	PC3	0	-0.191	-0.113	6440
5508	acemetacin	10 µM	HL60	0	-0.192	-0.207	2411
5509	mefexamide	13 µM	PC3	0	-0.192	-0.118	2121
5510	ramifenazone	14 µM	HL60	0	-0.192	-0.209	2534
5511	strophanthidin	10 µM	MCF7	0	-0.192	-0.163	7182
5512	nicardipine	8 µM	HL60	0	-0.192	-0.18	1600
5513	clofazimine	8 µM	PC3	0	-0.192	-0.145	4682
5514	fenofibrate	11 µM	MCF7	0	-0.192	-0.2	7432
5515	quipazine	9 µM	MCF7	0	-0.193	-0.23	2782
5516	pizotifen	9 µM	HL60	0	-0.193	-0.167	3134
5517	rilmenidine	8 µM	HL60	0	-0.193	-0.2	3133
5518	tetracycline	8 µM	MCF7	0	-0.193	-0.209	6233
5519	haloperidol	10 µM	MCF7	0	-0.193	-0.22	6960
5520	merbromin	5 µM	MCF7	0	-0.193	-0.213	3439
5521	carisoprodol	15 µM	HL60	0	-0.193	-0.215	1314
5522	benzathine benzylpenicillin	4 µM	MCF7	0	-0.193	-0.209	7359

5523	octopamine	21 µM	HL60	0	-0.193	-0.119	3112
5524	haloperidol	10 µM	MCF7	0	-0.194	-0.175	1024
5525	vanoxerine	8 µM	PC3	0	-0.194	-0.098	4641
5526	estradiol	10 nM	MCF7	0	-0.194	-0.206	6957
5527	pilocarpine	15 µM	MCF7	0	-0.194	-0.172	5341
5528	vigabatrin	31 µM	HL60	0	-0.194	-0.19	2452
5529	ergocalciferol	10 µM	MCF7	0	-0.194	-0.177	5346
5530	hexylcaine	13 µM	HL60	0	-0.195	-0.169	2708
5531	tinidazole	16 µM	MCF7	0	-0.195	-0.209	3896
5532	fluoxetine	12 µM	HL60	0	-0.195	-0.191	2453
5533	PHA-00745360	1 µM	PC3	0	-0.195	-0.159	4562
5534	tiaprofenic acid	15 µM	MCF7	0	-0.195	-0.219	2852
5535	mebeverine	9 µM	PC3	0	-0.195	-0.118	7147
5536	sulfadimethoxine	13 µM	MCF7	0	-0.195	-0.204	4724
5537	adiphenine	11 µM	MCF7	0	-0.195	-0.208	7037
5538	thapsigargin	100 nM	PC3	0	-0.195	-0.146	7103
5539	guanabenz	14 µM	PC3	0	-0.195	-0.14	4642
5540	genistein	10 µM	HL60	0	-0.195	-0.207	1176
5541	isometheptene	8 µM	HL60	0	-0.195	-0.198	3145
5542	3-hydroxy-DL-kynurenine	18 µM	HL60	0	-0.195	-0.225	1300
5543	methanthelinium bromide	10 µM	HL60	0	-0.195	-0.197	6137
5544	eticlopride	11 µM	HL60	0	-0.195	-0.156	3056
5545	telenzepine	9 µM	MCF7	0	-0.196	-0.194	7419
5546	merbromin	5 µM	PC3	0	-0.196	-0.071	3700
5547	alprostadil	10 µM	MCF7	0	-0.196	-0.205	6576
5548	lisuride	12 µM	MCF7	0	-0.196	-0.206	1545
5549	genistein	10 µM	MCF7	0	-0.196	-0.207	6952
5550	co-dergocrine mesilate	6 µM	MCF7	0	-0.196	-0.193	2793
5551	prazosin	10 µM	PC3	0	-0.196	-0.115	6315
5552	thiamphenicol	11 µM	PC3	0	-0.196	-0.112	7274
5553	convolamine	12 µM	MCF7	0	-0.196	-0.23	2771
5554	lactobionic acid	11 µM	HL60	0	-0.196	-0.193	1309
5555	meteneprost	10 µM	PC3	0	-0.197	-0.139	7504
5556	pirenperone	10 µM	PC3	0	-0.197	-0.121	4679
5557	tretinoin	1 µM	MCF7	0	-0.197	-0.239	849
5558	carteolol	12 µM	MCF7	0	-0.197	-0.203	3276
5559	terconazole	8 µM	PC3	0	-0.197	-0.133	4583
5560	disopyramide	12 µM	PC3	0	-0.197	-0.141	7276

5561	stachydrine	22 µM	MCF7	0	-0.198	-0.218	2743
5562	acemetacin	10 µM	MCF7	0	-0.198	-0.243	7442
5563	carbarsone	15 µM	HL60	0	-0.198	-0.238	1313
5564	estradiol	10 nM	MCF7	0	-0.198	-0.199	7000
5565	amitriptyline	13 µM	PC3	0	-0.198	-0.099	1823
5566	terazosin	9 µM	MCF7	0	-0.198	-0.181	7187
5567	valproic acid	500 µM	MCF7	0	-0.198	-0.168	6999
5568	estradiol	10 nM	HL60	0	-0.198	-0.173	1182
5569	moracizine	9 µM	MCF7	0	-0.198	-0.111	6000
5570	atracurium besilate	3 µM	PC3	0	-0.198	-0.116	1824
5571	chlorpropamide	100 µM	MCF7	0	-0.198	-0.166	144
5572	alexidine	7 µM	HL60	0	-0.199	-0.238	2576
5573	nitrofural	20 µM	HL60	0	-0.199	-0.2	2459
5574	Gly-His-Lys	1 µM	PC3	0	-0.199	-0.108	6570
5575	tretinoin	1 µM	HL60	0	-0.199	-0.172	6170
5576	ajmaline	12 µM	MCF7	0	-0.199	-0.175	7484
5577	betulinic acid	9 µM	MCF7	0	-0.199	-0.196	3281
5578	myricetin	13 µM	HL60	0	-0.2	-0.169	1334
5579	dosulepin	12 µM	HL60	0	-0.2	-0.156	1713
5580	ifosfamide	15 µM	MCF7	0	-0.2	-0.2	3485
5581	alprenolol	14 µM	PC3	0	-0.2	-0.139	7141
5582	iopromide	5 µM	PC3	0	-0.2	-0.126	4504
5583	hexetidine	12 µM	HL60	0	-0.2	-0.209	2457
5584	5186223	12 µM	MCF7	0	-0.2	-0.233	885
5585	loracarbef	11 µM	HL60	0	-0.2	-0.249	2970
5586	tranexamic acid	25 µM	PC3	0	-0.2	-0.123	2085
5587	chlorogenic acid	11 µM	PC3	0	-0.2	-0.141	4024
5588	erythromycin	5 µM	MCF7	0	-0.201	-0.172	5329
5589	streptozocin	15 µM	MCF7	0	-0.201	-0.202	7193
5590	mebhydrolin	5 µM	HL60	0	-0.201	-0.207	1333
5591	meclofenamic acid	12 µM	PC3	0	-0.201	-0.109	2128
5592	lidocaine	15 µM	MCF7	0	-0.201	-0.194	4421
5593	acetazolamide	18 µM	PC3	0	-0.201	-0.14	1808
5594	levopropoxyphene	7 µM	MCF7	0	-0.201	-0.18	3543
5595	fursultiamine	9 µM	MCF7	0	-0.201	-0.192	7349
5596	succinylsulfathiazole	11 µM	MCF7	0	-0.201	-0.185	2821
5597	bethanechol	20 µM	PC3	0	-0.201	-0.108	5114
5598	ceforanide	8 µM	MCF7	0	-0.201	-0.178	5351

5599	hexylcaine	13 µM	MCF7	0	-0.201	-0.183	6244
5600	primidone	18 µM	HL60	0	-0.201	-0.179	3065
5601	trihexyphenidyl	12 µM	HL60	0	-0.201	-0.187	2158
5602	5186324	2 µM	MCF7	0	-0.201	-0.198	900
5603	Prestwick-1103	20 µM	PC3	0	-0.202	-0.13	7317
5604	meclocycline	6 µM	HL60	0	-0.202	-0.218	1341
5605	tetryzoline	17 µM	MCF7	0	-0.202	-0.162	6769
5606	khellin	15 µM	PC3	0	-0.202	-0.116	6641
5607	paromomycin	6 µM	HL60	0	-0.202	-0.168	3017
5608	ciclosporin	3 µM	PC3	0	-0.202	-0.145	4586
5609	mimosine	20 µM	MCF7	0	-0.202	-0.216	5302
5610	metoclopramide	12 µM	PC3	0	-0.202	-0.132	3728
5611	haloperidol	10 µM	HL60	0	-0.202	-0.214	1185
5612	chlorprothixene	11 µM	MCF7	0	-0.202	-0.234	5291
5613	pepstatin	6 µM	MCF7	0	-0.202	-0.19	3264
5614	Prestwick-642	14 µM	MCF7	0	-0.203	-0.213	2815
5615	enoxacin	12 µM	HL60	0	-0.203	-0.22	1597
5616	(+/-)-catechin	14 µM	HL60	0	-0.203	-0.169	3012
5617	mefenamic acid	17 µM	HL60	0	-0.203	-0.229	1863
5618	valproic acid	500 µM	MCF7	0	-0.203	-0.194	1665
5619	midodrine	14 µM	PC3	0	-0.203	-0.117	7156
5620	tolmetin	13 µM	HL60	0	-0.203	-0.191	3009
5621	meteneprost	10 µM	MCF7	0	-0.203	-0.21	7552
5622	piperine	14 µM	HL60	0	-0.204	-0.187	1327
5623	dyclonine	12 µM	MCF7	0	-0.204	-0.195	7423
5624	CP-690334-01	1 µM	PC3	0	-0.204	-0.207	4561
5625	letrozole	14 µM	MCF7	0	-0.204	-0.213	4824
5626	methyldopa	19 µM	PC3	0	-0.204	-0.133	4677
5627	dicloxacillin	8 µM	MCF7	0	-0.204	-0.165	5012
5628	arecoline	17 µM	PC3	0	-0.205	-0.127	6322
5629	iopamidol	5 µM	MCF7	0	-0.205	-0.185	7189
5630	cyclobenzaprine	13 µM	HL60	0	-0.205	-0.21	1332
5631	sulfamonomethoxine	14 µM	MCF7	0	-0.205	-0.213	7200
5632	vinblastine	100 nM	MCF7	0	-0.205	-0.2	7551
5633	trimipramine	10 µM	HL60	0	-0.205	-0.172	3004
5634	metampicillin	10 µM	PC3	0	-0.205	-0.104	2123
5635	pergolide	10 µM	PC3	0	-0.205	-0.13	7271
5636	doxycycline	8 µM	MCF7	0	-0.205	-0.203	7195

5637	cefoperazone	6 µM	PC3	0	-0.205	-0.104	6323
5638	fulvestrant	1 µM	PC3	0	-0.205	-0.124	7539
5639	Prestwick-682	6 µM	HL60	0	-0.205	-0.228	2164
5640	isosorbide	17 µM	PC3	0	-0.206	-0.108	3720
5641	atractyloside	5 µM	HL60	0	-0.206	-0.212	2573
5642	lumicolchicine	10 µM	HL60	0	-0.206	-0.21	1317
5643	cefotiam	7 µM	MCF7	0	-0.206	-0.198	5361
5644	CP-863187	10 µM	MCF7	0	-0.206	-0.232	7553
5645	saquinavir	5 µM	HL60	0	-0.206	-0.159	6127
5646	pentoxifylline	14 µM	PC3	0	-0.206	-0.118	7319
5647	estradiol	10 nM	MCF7	0	-0.207	-0.222	1666
5648	nystatin	4 µM	HL60	0	-0.207	-0.175	2500
5649	geldanamycin	1 µM	MCF7	0	-0.207	-0.208	864
5650	iohexol	5 µM	PC3	0	-0.207	-0.126	4643
5651	monorden	100 nM	HL60	0	-0.207	-0.18	2679
5652	isotretinoin	13 µM	MCF7	0	-0.207	-0.196	7438
5653	alpha-ergocryptine	7 µM	HL60	0	-0.207	-0.215	2572
5654	prochlorperazine	7 µM	HL60	0	-0.207	-0.22	1286
5655	Prestwick-692	7 µM	HL60	0	-0.207	-0.2	2165
5656	penbutolol	6 µM	HL60	0	-0.207	-0.227	2972
5657	rosiglitazone	10 µM	MCF7	0	-0.208	-0.232	1071
5658	dapsone	16 µM	PC3	0	-0.208	-0.164	1827
5659	primaquine	9 µM	HL60	0	-0.209	-0.187	1343
5660	carbenoxolone	7 µM	HL60	0	-0.209	-0.188	3014
5661	cyclopenthiazide	11 µM	HL60	0	-0.209	-0.23	2905
5662	ketotifen	9 µM	MCF7	0	-0.209	-0.186	7199
5663	brinzolamide	10 µM	MCF7	0	-0.209	-0.219	5016
5664	azacyclonol	15 µM	MCF7	0	-0.209	-0.188	1520
5665	minocycline	11 µM	MCF7	0	-0.209	-0.195	1135
5666	noscapine	10 µM	MCF7	0	-0.21	-0.201	2745
5667	hydroxyachillin	14 µM	HL60	0	-0.21	-0.244	2157
5668	genistein	10 µM	MCF7	0	-0.21	-0.209	1660
5669	calycanthine	12 µM	MCF7	0	-0.21	-0.195	6221
5670	androsterone	14 µM	PC3	0	-0.211	-0.093	4635
5671	cefotaxime	8 µM	MCF7	0	-0.211	-0.185	7186
5672	vitexin	9 µM	HL60	0	-0.211	-0.223	2155
5673	methyldopate	15 µM	MCF7	0	-0.211	-0.208	7360
5674	naftifine	12 µM	PC3	0	-0.211	-0.129	7273

5675	pyrithyldione	24 µM	PC3	0	-0.211	-0.131	7153
5676	bupivacaine	12 µM	MCF7	0	-0.212	-0.192	7435
5677	sulfadimethoxine	13 µM	HL60	0	-0.212	-0.224	2578
5678	tolnaftate	13 µM	MCF7	0	-0.212	-0.224	1501
5679	tacrine	16 µM	MCF7	0	-0.212	-0.211	5297
5680	dapsone	16 µM	MCF7	0	-0.212	-0.218	1705
5681	Prestwick-984	9 µM	HL60	0	-0.212	-0.213	2903
5682	timolol	9 µM	PC3	0	-0.212	-0.147	4685
5683	pentoxifylline	14 µM	PC3	0	-0.213	-0.138	2127
5684	neostigmine bromide	13 µM	MCF7	0	-0.213	-0.221	5335
5685	naproxen	17 µM	PC3	0	-0.213	-0.125	6358
5686	streptomycin	3 µM	MCF7	0	-0.213	-0.247	7194
5687	sulindac	11 µM	PC3	0	-0.213	-0.137	5103
5688	halofantrine	7 µM	HL60	0	-0.214	-0.231	3130
5689	benzocaine	24 µM	HL60	0	-0.214	-0.208	2167
5690	triamterene	16 µM	PC3	0	-0.215	-0.116	1819
5691	Gly-His-Lys	1 µM	MCF7	0	-0.215	-0.214	6575
5692	prednisone	11 µM	PC3	0	-0.215	-0.132	4577
5693	heptaminol	22 µM	PC3	0	-0.215	-0.13	1825
5694	Prestwick-692	7 µM	PC3	0	-0.215	-0.138	4599
5695	homatropine	11 µM	MCF7	0	-0.215	-0.232	1684
5696	PF-00539758-00	10 µM	PC3	0	-0.216	-0.137	6421
5697	meclofenamic acid	12 µM	MCF7	0	-0.216	-0.18	7038
5698	dapsone	16 µM	HL60	0	-0.216	-0.204	1868
5699	propidium iodide	6 µM	HL60	0	-0.216	-0.181	2541
5700	epirizole	17 µM	PC3	0	-0.216	-0.156	1803
5701	procarbazine	16 µM	HL60	0	-0.217	-0.23	2971
5702	nifedipine	12 µM	PC3	0	-0.217	-0.161	1814
5703	isoniazid	29 µM	MCF7	0	-0.217	-0.194	7197
5704	lansoprazole	11 µM	HL60	0	-0.217	-0.269	2967
5705	clozapine	10 µM	HL60	0	-0.217	-0.191	1170
5706	xamoterol	5 µM	MCF7	0	-0.218	-0.209	5363
5707	gentamicin	3 µM	PC3	0	-0.218	-0.112	5883
5708	thiamphenicol	11 µM	PC3	0	-0.218	-0.149	1826
5709	estradiol	10 nM	HL60	0	-0.218	-0.234	2701
5710	ondansetron	12 µM	HL60	0	-0.219	-0.201	6153
5711	aconitine	6 µM	PC3	0	-0.219	-0.168	7149
5712	DL-PPMP	2 µM	MCF7	0	-0.219	-0.236	1121

5713	diphenhydramine	14 µM	PC3	0	-0.219	-0.124	1830
5714	viomycin	5 µM	PC3	0	-0.219	-0.165	7278
5715	dexibuprofen	19 µM	HL60	0	-0.219	-0.213	3094
5716	levopropoxyphene	7 µM	HL60	0	-0.219	-0.249	2980
5717	adiphenine	11 µM	PC3	0	-0.219	-0.143	1831
5718	cefmetazole	8 µM	HL60	0	-0.22	-0.193	2524
5719	Prestwick-691	14 µM	MCF7	0	-0.22	-0.238	2813
5720	merbromin	5 µM	HL60	0	-0.22	-0.21	2577
5721	BCB000040	10 µM	MCF7	0	-0.22	-0.254	7554
5722	thiamazole	35 µM	HL60	0	-0.22	-0.194	2570
5723	propoxycaine	12 µM	PC3	0	-0.22	-0.15	7155
5724	cefotiam	7 µM	HL60	0	-0.221	-0.183	2458
5725	ambroxol	10 µM	MCF7	0	-0.221	-0.243	5319
5726	tyrphostin AG-1478	32 µM	MCF7	0	-0.222	-0.242	1141
5727	Chicago Sky Blue 6B	4 µM	HL60	0	-0.223	-0.227	1330
5728	pyrithyldione	24 µM	MCF7	0	-0.224	-0.226	3482
5729	naproxen	17 µM	MCF7	0	-0.224	-0.208	1706
5730	nadide	6 µM	HL60	0	-0.224	-0.192	2529
5731	clemastine	9 µM	MCF7	0	-0.224	-0.23	7443
5732	chlorpromazine	11 µM	HL60	0	-0.224	-0.246	1864
5733	isoflupredone	10 µM	HL60	0	-0.224	-0.24	1873
5734	naftidrofuryl	8 µM	MCF7	0	-0.224	-0.235	5287
5735	sirolimus	100 nM	HL60	0	-0.224	-0.247	1183
5736	heptaminol	22 µM	HL60	0	-0.225	-0.199	1866
5737	harpagoside	8 µM	MCF7	0	-0.225	-0.231	7355
5738	flucloxacillin	8 µM	HL60	0	-0.226	-0.183	3128
5739	dl-alpha tocopherol	9 µM	HL60	0	-0.226	-0.234	1320
5740	diethylcarbamazine	10 µM	MCF7	0	-0.226	-0.239	7425
5741	alprostadil	11 µM	MCF7	0	-0.227	-0.213	7358
5742	dimenhydrinate	9 µM	MCF7	0	-0.228	-0.211	7431
5743	corticosterone	12 µM	HL60	0	-0.228	-0.23	1307
5744	haloperidol	10 µM	MCF7	0	-0.228	-0.238	1082
5745	clozapine	12 µM	HL60	0	-0.228	-0.249	1289
5746	vinburnine	14 µM	PC3	0	-0.229	-0.175	7154
5747	3-acetamidocoumarin	20 µM	MCF7	0	-0.229	-0.247	7361
5748	SR-95639A	10 µM	HL60	0	-0.229	-0.237	1336
5749	betulin	9 µM	HL60	0	-0.231	-0.237	2952
5750	isoflupredone	10 µM	PC3	0	-0.232	-0.171	1832

5751	asiaticoside	4 µM	HL60	0	-0.232	-0.22	2943
5752	bumetanide	11 µM	MCF7	0	-0.232	-0.256	7440
5753	streptozocin	15 µM	HL60	0	-0.233	-0.24	2535
5754	fulvestrant	10 nM	HL60	0	-0.235	-0.233	1179
5755	podophyllotoxin	10 µM	MCF7	0	-0.235	-0.202	7198
5756	cyclic adenosine monophosphate	12 µM	HL60	0	-0.236	-0.245	2969
5757	chlortalidone	12 µM	PC3	0	-0.236	-0.179	7152
5758	diprophylline	16 µM	HL60	0	-0.236	-0.173	1853
5759	enalapril	8 µM	MCF7	0	-0.238	-0.235	7428
5760	viomycin	5 µM	HL60	0	-0.238	-0.223	2979
5761	adiphenine	11 µM	PC3	0	-0.239	-0.132	7279
5762	azapropazone	13 µM	PC3	0	-0.24	-0.168	7277
5763	estradiol	10 nM	MCF7	0	-0.24	-0.214	1021
5764	chlorogenic acid	11 µM	HL60	0	-0.242	-0.252	1346
5765	ozagrel	15 µM	HL60	0	-0.242	-0.257	2942
5766	boldine	12 µM	HL60	0	-0.246	-0.199	2148
5767	proguanil	14 µM	HL60	0	-0.249	-0.234	2944
5768	homatropine	11 µM	HL60	0	-0.25	-0.219	1848
5769	diphenhydramine	14 µM	HL60	0	-0.251	-0.262	1871
5770	adiphenine	11 µM	HL60	0	-0.261	-0.275	1872
5771	metformin	24 µM	HL60	0	-0.262	-0.256	1858
5772	NU-1025	100 µM	MCF7	-0.436	-0.066	0.072	313
5773	iloprost	1 µM	SKMEL5	-0.47	-0.1	0.048	496
5774	exisulind	50 µM	MCF7	-0.473	-0.068	0.082	314
5775	tolnaftate	13 µM	PC3	-0.474	-0.084	0.066	4221
5776	chlorhexidine	8 µM	PC3	-0.475	-0.107	0.043	1942
5777	raloxifene	100 nM	HL60	-0.475	-0.078	0.073	388
5778	monastrol	20 µM	MCF7	-0.477	-0.075	0.076	614
5779	0173570-0000	1 µM	PC3	-0.477	-0.096	0.055	3693
5780	betonicine	25 µM	PC3	-0.486	-0.106	0.047	4301
5781	ciclosporin	1 µM	MCF7	-0.486	-0.066	0.088	261
5782	dicloxacillin	8 µM	PC3	-0.49	-0.086	0.069	6666
5783	SC-58125	10 µM	SKMEL5	-0.495	-0.075	0.081	507
5784	Prestwick-674	14 µM	PC3	-0.501	-0.1	0.058	3716
5785	novobiocin	100 µM	MCF7	-0.514	-0.083	0.08	342
5786	thioridazine	10 µM	MCF7	-0.515	-0.067	0.096	5916
5787	tanespimycin	1 µM	PC3	-0.516	-0.1	0.064	4450

5788	karakoline	11 µM	PC3	-0.518	-0.091	0.073	4297
5789	tetraethylenepentamine	100 µM	PC3	-0.52	-0.097	0.068	457
5790	ketoprofen	16 µM	PC3	-0.525	-0.099	0.067	3729
5791	sirolimus	100 nM	PC3	-0.526	-0.12	0.047	4466
5792	carbenoxolone	7 µM	MCF7	-0.528	-0.072	0.095	4173
5793	PF-00562151-00	10 µM	MCF7	-0.53	-0.078	0.09	5954
5794	mexiletine	19 µM	MCF7	-0.53	-0.069	0.099	3973
5795	metrizamide	5 µM	MCF7	-0.531	-0.092	0.076	4156
5796	tanespimycin	1 µM	MCF7	-0.533	-0.095	0.074	381
5797	mebeverine	9 µM	MCF7	-0.535	-0.098	0.072	3193
5798	labetalol	11 µM	PC3	-0.535	-0.108	0.061	4473
5799	diclofenac	10 µM	MCF7	-0.539	-0.082	0.088	333
5800	ciclopirox	15 µM	PC3	-0.541	-0.098	0.073	6677
5801	diperodon	9 µM	PC3	-0.541	-0.102	0.069	4498
5802	clindamycin	9 µM	HL60	-0.542	-0.084	0.088	1373
5803	triamcinolone	10 µM	MCF7	-0.543	-0.09	0.082	2241
5804	vincamine	11 µM	MCF7	-0.544	-0.088	0.084	3865
5805	sulfamethizole	15 µM	PC3	-0.545	-0.098	0.074	5798
5806	dexamethasone	1 µM	MCF7	-0.549	-0.073	0.101	255
5807	xylazine	18 µM	PC3	-0.55	-0.122	0.052	4066
5808	fendiline	11 µM	MCF7	-0.554	-0.106	0.07	3190
5809	NU-1025	100 µM	MCF7	-0.554	-0.093	0.082	608
5810	famprofazone	11 µM	MCF7	-0.554	-0.089	0.086	3834
5811	quinpirole	1 µM	MCF7	-0.556	-0.099	0.077	456
5812	trimethadione	28 µM	PC3	-0.558	-0.082	0.095	4086
5813	bendroflumethiazide	9 µM	PC3	-0.558	-0.109	0.067	4315
5814	famotidine	12 µM	PC3	-0.56	-0.108	0.069	1946
5815	tiratricol	6 µM	MCF7	-0.56	-0.088	0.09	2259
5816	diphemanil metilsulfate	10 µM	PC3	-0.561	-0.108	0.069	1912
5817	citiolone	25 µM	MCF7	-0.565	-0.081	0.097	3836
5818	dioxybenzone	16 µM	PC3	-0.565	-0.116	0.063	4638
5819	azathioprine	14 µM	PC3	-0.565	-0.117	0.062	1945
5820	lidocaine	15 µM	PC3	-0.566	-0.105	0.074	1917
5821	nystatin	4 µM	PC3	-0.567	-0.111	0.068	4223
5822	ethisterone	13 µM	MCF7	-0.569	-0.08	0.1	3975
5823	perphenazine	10 µM	PC3	-0.57	-0.116	0.064	4637
5824	meclocycline	6 µM	PC3	-0.573	-0.108	0.073	6637
5825	propofol	22 µM	MCF7	-0.573	-0.096	0.085	3386

5826	metergoline	10 µM	PC3	-0.574	-0.118	0.064	6744
5827	tanespimycin	1 µM	MCF7	-0.574	-0.09	0.092	5914
5828	mesalazine	26 µM	PC3	-0.575	-0.095	0.087	5888
5829	W-13	10 µM	MCF7	-0.576	-0.09	0.092	643
5830	gibberellic acid	12 µM	PC3	-0.576	-0.103	0.079	4234
5831	amprolium	13 µM	PC3	-0.577	-0.113	0.07	1898
5832	oxantel	7 µM	PC3	-0.577	-0.091	0.091	6738
5833	probucol	10 µM	MCF7	-0.578	-0.104	0.079	592
5834	bucladesine	8 µM	HL60	-0.582	-0.096	0.088	2741
5835	geldanamycin	1 µM	PC3	-0.582	-0.117	0.067	4452
5836	norfloxacin	13 µM	MCF7	-0.586	-0.09	0.095	2253
5837	ursolic acid	9 µM	PC3	-0.586	-0.109	0.076	5825
5838	nifedipine	10 µM	MCF7	-0.587	-0.102	0.084	603
5839	phenformin	10 µM	MCF7	-0.587	-0.097	0.088	21
5840	meclofenoxate	14 µM	MCF7	-0.588	-0.093	0.093	4729
5841	torasemide	11 µM	PC3	-0.59	-0.104	0.083	5057
5842	hydrochlorothiazide	13 µM	PC3	-0.59	-0.12	0.067	1906
5843	praziquantel	13 µM	MCF7	-0.594	-0.107	0.081	3189
5844	thiethylperazine	6 µM	PC3	-0.595	-0.133	0.055	5756
5845	proscillaridin	8 µM	MCF7	-0.595	-0.094	0.095	4404
5846	helveticoside	7 µM	MCF7	-0.599	-0.089	0.1	6047
5847	cicloheximide	14 µM	PC3	-0.599	-0.107	0.082	5743
5848	oleandomycin	5 µM	PC3	-0.599	-0.121	0.068	1935
5849	spiramycin	5 µM	MCF7	-0.6	-0.085	0.105	3844
5850	tetrandrine	6 µM	PC3	-0.6	-0.111	0.079	5821
5851	metixene	12 µM	PC3	-0.601	-0.089	0.101	6672
5852	calmidazolium	5 µM	MCF7	-0.602	-0.109	0.081	486
5853	loxapine	9 µM	PC3	-0.603	-0.108	0.082	6694
5854	probucol	8 µM	MCF7	-0.604	-0.088	0.103	3223
5855	tanespimycin	1 µM	PC3	-0.608	-0.113	0.079	5958
5856	proxymetacaine	12 µM	MCF7	-0.608	-0.112	0.08	5433
5857	altizide	10 µM	MCF7	-0.609	-0.109	0.084	6089
5858	protriptyline	13 µM	MCF7	-0.609	-0.094	0.099	6498
5859	N6-methyladenosine	14 µM	PC3	-0.609	-0.139	0.054	6732
5860	levodopa	20 µM	PC3	-0.611	-0.124	0.069	4571
5861	metitepine	8 µM	MCF7	-0.612	-0.087	0.107	5413
5862	mefloquine	10 µM	MCF7	-0.612	-0.082	0.112	2210
5863	trifluoperazine	10 µM	HL60	-0.613	-0.103	0.091	2684

5864	mafenide	18 µM	MCF7	-0.615	-0.093	0.101	5499
5865	cinchonidine	14 µM	PC3	-0.615	-0.105	0.089	5833
5866	suloctidil	12 µM	PC3	-0.615	-0.1	0.095	6675
5867	isometheptene	8 µM	MCF7	-0.616	-0.099	0.096	6524
5868	azacyclonol	15 µM	MCF7	-0.616	-0.097	0.098	5398
5869	phensuximide	21 µM	PC3	-0.616	-0.121	0.074	5097
5870	pempidine	13 µM	MCF7	-0.621	-0.103	0.094	3832
5871	felodipine	10 µM	PC3	-0.621	-0.112	0.084	6695
5872	levomepromazine	9 µM	PC3	-0.622	-0.117	0.08	7399
5873	ascorbic acid	22 µM	MCF7	-0.622	-0.087	0.11	3225
5874	ritodrine	12 µM	PC3	-0.623	-0.127	0.071	4619
5875	terguride	9 µM	PC3	-0.624	-0.111	0.086	4633
5876	mianserin	13 µM	MCF7	-0.624	-0.099	0.098	2231
5877	MK-886	1 µM	MCF7	-0.628	-0.117	0.082	601
5878	chlorpromazine	1 µM	MCF7	-0.629	-0.124	0.075	426
5879	ondansetron	12 µM	MCF7	-0.631	-0.102	0.098	3575
5880	ciprofloxacin	11 µM	PC3	-0.631	-0.125	0.074	6700
5881	16,16-dimethylprostaglandin E2	10 µM	PC3	-0.632	-0.111	0.089	6562
5882	sulpiride	12 µM	MCF7	-0.634	-0.088	0.113	4389
5883	trapidil	19 µM	MCF7	-0.634	-0.106	0.095	6515
5884	Prestwick-1100	9 µM	MCF7	-0.634	-0.102	0.098	3880
5885	tretinoin	1 µM	PC3	-0.636	-0.132	0.069	1211
5886	scopolamine	12 µM	MCF7	-0.637	-0.096	0.105	4803
5887	orlistat	10 µM	MCF7	-0.637	-0.076	0.125	6383
5888	CP-319743	10 µM	PC3	-0.638	-0.121	0.08	7537
5889	paclitaxel	5 µM	MCF7	-0.638	-0.115	0.087	1542
5890	furaltadone	11 µM	HL60	-0.639	-0.104	0.098	2554
5891	methylbenzethonium chloride	9 µM	MCF7	-0.64	-0.107	0.095	3850
5892	hydrocotarnine	13 µM	PC3	-0.642	-0.123	0.08	4489
5893	danazol	12 µM	PC3	-0.644	-0.108	0.095	1954
5894	econazole	9 µM	PC3	-0.644	-0.127	0.077	7305
5895	megestrol	10 µM	MCF7	-0.646	-0.103	0.101	6468
5896	butyl hydroxybenzoate	21 µM	MCF7	-0.646	-0.1	0.104	5608
5897	midecamycin	5 µM	PC3	-0.647	-0.104	0.101	1943
5898	proguanil	14 µM	MCF7	-0.648	-0.109	0.096	3505
5899	phentolamine	13 µM	MCF7	-0.648	-0.103	0.102	2323

5900	urapidil	9 µM	PC3	-0.649	-0.127	0.078	6696
5901	diprophylline	16 µM	MCF7	-0.65	-0.104	0.101	5482
5902	aciclovir	18 µM	PC3	-0.652	-0.125	0.081	1960
5903	tenoxicam	12 µM	PC3	-0.652	-0.15	0.056	4102
5904	allantoin	25 µM	MCF7	-0.652	-0.084	0.122	5471
5905	josamycin	5 µM	PC3	-0.653	-0.117	0.089	1950
5906	clorsulon	11 µM	HL60	-0.655	-0.119	0.089	1735
5907	puromycin	7 µM	MCF7	-0.656	-0.095	0.113	3310
5908	bucladesine	20 µM	MCF7	-0.656	-0.112	0.096	591
5909	dextromethorphan	11 µM	MCF7	-0.658	-0.105	0.104	5401
5910	ivermectin	5 µM	PC3	-0.658	-0.103	0.105	5853
5911	pindolol	16 µM	MCF7	-0.658	-0.106	0.102	6834
5912	SC-19220	10 µM	PC3	-0.659	-0.132	0.076	7065
5913	tanespimycin	1 µM	HL60	-0.66	-0.103	0.106	2666
5914	paclitaxel	5 µM	PC3	-0.66	-0.112	0.097	1959
5915	salsolinol	15 µM	PC3	-0.66	-0.102	0.107	4232
5916	chlorhexidine	8 µM	PC3	-0.661	-0.125	0.084	6302
5917	nefopam	14 µM	MCF7	-0.661	-0.087	0.122	4752
5918	flupentixol	8 µM	MCF7	-0.662	-0.106	0.103	2643
5919	pizotifen	9 µM	MCF7	-0.664	-0.092	0.118	5491
5920	PF-00562151-00	10 µM	PC3	-0.665	-0.11	0.1	6863
5921	diprophylline	16 µM	PC3	-0.665	-0.143	0.067	5063
5922	ketoconazole	8 µM	MCF7	-0.665	-0.1	0.111	2640
5923	orphenadrine	13 µM	MCF7	-0.667	-0.088	0.123	3883
5924	flumequine	15 µM	MCF7	-0.668	-0.11	0.102	5529
5925	apomorphine	6 µM	PC3	-0.668	-0.122	0.09	6683
5926	mianserin	13 µM	PC3	-0.67	-0.117	0.095	5786
5927	leflunomide	15 µM	MCF7	-0.67	-0.117	0.095	7238
5928	ciprofloxacin	11 µM	PC3	-0.671	-0.135	0.078	1939
5929	terbutaline	7 µM	PC3	-0.671	-0.138	0.075	5764
5930	carbenoxolone	7 µM	MCF7	-0.672	-0.122	0.091	3353
5931	altretamine	19 µM	PC3	-0.672	-0.108	0.104	4627
5932	mesoridazine	7 µM	PC3	-0.673	-0.124	0.089	7256
5933	tanespimycin	1 µM	PC3	-0.674	-0.143	0.07	1218
5934	emetine	7 µM	MCF7	-0.675	-0.111	0.103	2801
5935	santonin	16 µM	MCF7	-0.677	-0.12	0.094	3877
5936	thioridazine	10 µM	HL60	-0.679	-0.084	0.131	2690
5937	sulfaquinoxaline	12 µM	MCF7	-0.68	-0.104	0.111	6788

5938	erythromycin	5 µM	PC3	-0.681	-0.113	0.103	1928
5939	lanatoside C	4 µM	MCF7	-0.682	-0.102	0.114	3852
5940	MG-262	100 nM	PC3	-0.683	-0.133	0.083	7068
5941	solanine	5 µM	MCF7	-0.683	-0.134	0.082	4166
5942	megestrol	10 µM	MCF7	-0.684	-0.111	0.105	5013
5943	securinine	18 µM	MCF7	-0.686	-0.121	0.096	6831
5944	cefotaxime	8 µM	PC3	-0.686	-0.134	0.083	2072
5945	alfadolone	10 µM	PC3	-0.686	-0.138	0.079	7262
5946	chloropyrazine	35 µM	HL60	-0.687	-0.113	0.104	6148
5947	niflumic acid	14 µM	MCF7	-0.687	-0.088	0.13	5490
5948	verapamil	8 µM	MCF7	-0.688	-0.107	0.111	5387
5949	isocarboxazid	17 µM	HL60	-0.688	-0.115	0.103	2562
5950	glafenine	10 µM	PC3	-0.688	-0.129	0.089	7257
5951	pirenzepine	9 µM	PC3	-0.689	-0.113	0.105	5872
5952	cyclobenzaprine	13 µM	MCF7	-0.693	-0.097	0.122	4834
5953	dicycloverine	12 µM	PC3	-0.694	-0.14	0.08	4581
5954	piromidic acid	14 µM	PC3	-0.694	-0.139	0.08	4575
5955	lasalocid	7 µM	MCF7	-0.695	-0.129	0.09	4985
5956	harmalol	15 µM	MCF7	-0.695	-0.1	0.12	2892
5957	dirithromycin	5 µM	MCF7	-0.696	-0.118	0.102	2863
5958	atropine oxide	12 µM	MCF7	-0.696	-0.114	0.107	6812
5959	withaferin A	1 µM	MCF7	-0.697	-0.104	0.117	3902
5960	diphenhydramine	14 µM	PC3	-0.7	-0.137	0.084	7318
5961	quipazine	9 µM	MCF7	-0.701	-0.115	0.106	7240
5962	strophanthidin	10 µM	PC3	-0.703	-0.119	0.103	5826
5963	SC-560	10 µM	PC3	-0.703	-0.142	0.08	6865
5964	perhexiline	10 µM	PC3	-0.703	-0.103	0.12	5081
5965	tetracycline	8 µM	HL60	-0.704	-0.112	0.11	1397
5966	butoconazole	8 µM	HL60	-0.705	-0.108	0.115	2427
5967	alverine	8 µM	MCF7	-0.706	-0.106	0.117	2273
5968	(-)-isoprenaline	16 µM	HL60	-0.706	-0.133	0.09	6149
5969	fulvestrant	1 µM	PC3	-0.708	-0.141	0.083	704
5970	alverine	8 µM	PC3	-0.708	-0.118	0.106	6345
5971	pirlindole	12 µM	HL60	-0.708	-0.11	0.114	3140
5972	triamterene	16 µM	PC3	-0.708	-0.149	0.075	7307
5973	nocodazole	13 µM	PC3	-0.709	-0.124	0.1	7145
5974	flunixin	8 µM	MCF7	-0.711	-0.102	0.123	4735
5975	clebopride	8 µM	PC3	-0.712	-0.136	0.089	6311

5976	rilmenidine	8 µM	MCF7	-0.713	-0.116	0.11	6512
5977	oxybenzone	18 µM	PC3	-0.714	-0.135	0.091	6309
5978	colchicine	10 µM	MCF7	-0.716	-0.092	0.135	5675
5979	vinburnine	14 µM	HL60	-0.717	-0.113	0.114	1788
5980	azathioprine	14 µM	MCF7	-0.717	-0.125	0.102	5262
5981	piretanide	11 µM	HL60	-0.718	-0.13	0.097	6144
5982	aminophenazone	17 µM	PC3	-0.718	-0.129	0.098	2060
5983	dipivefrine	10 µM	MCF7	-0.722	-0.107	0.121	2744
5984	antazoline	13 µM	HL60	-0.723	-0.118	0.11	1556
5985	orciprenaline	8 µM	MCF7	-0.724	-0.091	0.138	4831
5986	neomycin	4 µM	PC3	-0.727	-0.122	0.108	5867
5987	geldanamycin	1 µM	MCF7	-0.727	-0.125	0.105	5225
5988	haloperidol	10 µM	MCF7	-0.728	-0.14	0.091	5604
5989	mafenide	18 µM	PC3	-0.728	-0.157	0.074	2124
5990	ticarcillin	9 µM	PC3	-0.73	-0.111	0.12	5829
5991	astemizole	9 µM	MCF7	-0.73	-0.105	0.126	6807
5992	digitoxigenin	11 µM	MCF7	-0.73	-0.129	0.102	4801
5993	tanespimycin	1 µM	MCF7	-0.732	-0.126	0.106	428
5994	sulpiride	12 µM	MCF7	-0.736	-0.108	0.125	1467
5995	harmine	16 µM	HL60	-0.736	-0.114	0.118	1758
5996	thiamine	12 µM	HL60	-0.737	-0.126	0.107	1744
5997	oxybenzone	18 µM	MCF7	-0.739	-0.116	0.118	5410
5998	acetylsalicylsalicylic acid	13 µM	MCF7	-0.742	-0.12	0.114	6778
5999	sulfapyridine	16 µM	PC3	-0.742	-0.132	0.102	7151
6000	sulfaguanidine	19 µM	MCF7	-0.743	-0.105	0.13	1495
6001	fulvestrant	1 µM	PC3	-0.744	-0.164	0.071	6867
6002	mianserin	13 µM	HL60	-0.745	-0.112	0.124	1385
6003	streptozocin	15 µM	PC3	-0.747	-0.16	0.076	5836
6004	pioglitazone	10 µM	MCF7	-0.748	-0.093	0.144	7523
6005	ramipril	10 µM	PC3	-0.748	-0.131	0.106	7144
6006	STOCK1N-28457	20 µM	MCF7	-0.749	-0.118	0.12	6869
6007	etodolac	14 µM	PC3	-0.75	-0.162	0.076	2091
6008	mebeverine	9 µM	HL60	-0.751	-0.122	0.115	1576
6009	amodiaquine	9 µM	HL60	-0.753	-0.126	0.112	1570
6010	tridihexethyl	11 µM	MCF7	-0.754	-0.131	0.108	5486
6011	chlorphenamine	10 µM	MCF7	-0.755	-0.094	0.145	6773
6012	pheneticillin	10 µM	MCF7	-0.757	-0.117	0.122	6239
6013	ranitidine	11 µM	HL60	-0.759	-0.123	0.117	1404

6014	clebopride	8 µM	MCF7	-0.759	-0.124	0.116	2646
6015	cephaeline	6 µM	HL60	-0.762	-0.14	0.101	2429
6016	fluspirilene	8 µM	MCF7	-0.762	-0.11	0.131	5008
6017	nicergoline	8 µM	PC3	-0.763	-0.156	0.086	2058
6018	metformin	24 µM	MCF7	-0.765	-0.144	0.098	5487
6019	hydroquinine	9 µM	HL60	-0.765	-0.129	0.113	1775
6020	nifurtimox	14 µM	MCF7	-0.765	-0.124	0.118	7328
6021	strophanthidin	10 µM	HL60	-0.767	-0.11	0.133	2525
6022	zomepirac	13 µM	MCF7	-0.768	-0.117	0.126	6815
6023	ethosuximide	28 µM	PC3	-0.769	-0.153	0.09	7308
6024	colforsin	500 nM	MCF7	-0.769	-0.111	0.132	7055
6025	16,16-dimethylprostaglandin E2	10 µM	MCF7	-0.769	-0.109	0.134	6597
6026	pilocarpine	15 µM	HL60	-0.77	-0.117	0.126	2438
6027	ampicillin	10 µM	MCF7	-0.77	-0.123	0.121	5408
6028	etifenin	12 µM	HL60	-0.771	-0.107	0.137	2477
6029	epivincamine	11 µM	MCF7	-0.772	-0.129	0.116	2775
6030	isoconazole	10 µM	HL60	-0.774	-0.118	0.127	1372
6031	chloropyramine	12 µM	PC3	-0.774	-0.118	0.127	4589
6032	piperacetazine	10 µM	HL60	-0.775	-0.144	0.101	6152
6033	suloctidil	12 µM	MCF7	-0.776	-0.144	0.102	2651
6034	suramin sodium	10 µM	MCF7	-0.778	-0.103	0.143	7496
6035	prenylamine	10 µM	MCF7	-0.78	-0.108	0.139	5489
6036	terazosin	9 µM	HL60	-0.78	-0.144	0.103	2530
6037	bumetanide	11 µM	HL60	-0.78	-0.135	0.112	2409
6038	nomifensine	11 µM	HL60	-0.781	-0.142	0.105	1378
6039	(-)-isoprenaline	16 µM	MCF7	-0.781	-0.103	0.144	3571
6040	ciclopirox	15 µM	HL60	-0.782	-0.122	0.126	2456
6041	cisapride	9 µM	HL60	-0.782	-0.112	0.136	2443
6042	lycorine	12 µM	HL60	-0.783	-0.127	0.121	2195
6043	bambuterol	10 µM	PC3	-0.784	-0.14	0.108	5885
6044	cefamandole	8 µM	PC3	-0.788	-0.128	0.122	7394
6045	demecarium bromide	6 µM	MCF7	-0.789	-0.149	0.1	2773
6046	alprostadil	11 µM	PC3	-0.79	-0.154	0.096	4099
6047	flucytosine	31 µM	HL60	-0.791	-0.115	0.136	3073
6048	digoxigenin	10 µM	MCF7	-0.792	-0.101	0.15	5275
6049	isoxicam	12 µM	MCF7	-0.792	-0.121	0.129	7028
6050	emetine	7 µM	HL60	-0.793	-0.161	0.09	2145

6051	guanethidine	13 µM	HL60	-0.794	-0.119	0.132	1554
6052	imipramine	13 µM	PC3	-0.796	-0.165	0.087	1807
6053	H-89	500 nM	MCF7	-0.801	-0.121	0.133	6878
6054	proadifen	10 µM	MCF7	-0.801	-0.117	0.136	3446
6055	dobutamine	12 µM	HL60	-0.805	-0.128	0.127	1589
6056	tamoxifen	7 µM	HL60	-0.808	-0.119	0.137	1366
6057	Prestwick-689	10 µM	MCF7	-0.811	-0.119	0.138	7173
6058	diphemanil metilsulfate	10 µM	MCF7	-0.814	-0.112	0.146	1494
6059	doxepin	13 µM	MCF7	-0.818	-0.13	0.129	7415
6060	anabasine	25 µM	MCF7	-0.819	-0.113	0.146	6774
6061	etamivan	18 µM	MCF7	-0.819	-0.123	0.136	7021
6062	cotinine	23 µM	HL60	-0.821	-0.137	0.123	2011
6063	hecogenin	9 µM	MCF7	-0.821	-0.128	0.132	7175
6064	clemastine	9 µM	MCF7	-0.823	-0.13	0.131	7485
6065	glipizide	9 µM	HL60	-0.825	-0.124	0.137	2008
6066	diprophylline	16 µM	MCF7	-0.826	-0.125	0.137	1689
6067	alimemazine	5 µM	MCF7	-0.826	-0.126	0.136	3478
6068	phenformin	17 µM	HL60	-0.828	-0.134	0.128	2350
6069	homochlorcyclizine	10 µM	PC3	-0.829	-0.135	0.127	7295
6070	artemisinin	14 µM	HL60	-0.83	-0.126	0.137	1714
6071	colchicine	10 µM	HL60	-0.83	-0.136	0.126	1598
6072	praziquantel	13 µM	HL60	-0.831	-0.133	0.13	1572
6073	lovastatin	10 µM	HL60	-0.836	-0.133	0.131	2494
6074	nadide	6 µM	MCF7	-0.841	-0.119	0.147	7227
6075	15-delta prostaglandin J2	10 µM	MCF7	-0.843	-0.136	0.131	6990
6076	metanephrine	17 µM	MCF7	-0.865	-0.138	0.136	5334
6077	mesoridazine	7 µM	MCF7	-0.866	-0.138	0.136	7017
6078	ciprofloxacin	11 µM	MCF7	-0.866	-0.141	0.133	5299
6079	proscillaridin	8 µM	MCF7	-0.871	-0.134	0.141	7340
6080	naltrexone	10 µM	MCF7	-0.873	-0.156	0.12	6241
6081	lanatoside C	4 µM	HL60	-0.876	-0.147	0.13	2193
6082	benzydamine	12 µM	MCF7	-0.877	-0.151	0.126	7169
6083	11-deoxy-16,16-dimethylprostaglandin E2	10 µM	PC3	-0.881	-0.16	0.118	7538
6084	monorden	100 nM	HL60	-0.884	-0.128	0.152	1160
6085	demecolcine	12 µM	MCF7	-0.885	-0.143	0.137	1103
6086	15-delta prostaglandin J2	10 µM	MCF7	-0.887	-0.147	0.134	1011
6087	sulfaphenazole	13 µM	MCF7	-0.895	-0.136	0.147	1673

6088	SR-95531	11 µM	HL60	-0.897	-0.146	0.138	1316
6089	thioridazine	10 µM	HL60	-0.898	-0.132	0.152	1171
6090	nitrofural	20 µM	MCF7	-0.91	-0.158	0.13	5321
6091	cytochalasin B	21 µM	MCF7	-0.917	-0.134	0.156	1122
6092	cinnarizine	11 µM	MCF7	-0.938	-0.156	0.141	7174
6093	N-acetylmuramic acid	14 µM	HL60	-0.946	-0.157	0.142	1326
6094	5162773	7 µM	MCF7	-0.947	-0.132	0.168	892
6095	dihydroergotamine	3 µM	PC3	-0.949	-0.221	0.079	2081
6096	bucladesine	2 µM	MCF7	-0.958	-0.132	0.171	842
6097	N-acetyl-L-aspartic acid	23 µM	HL60	-0.958	-0.148	0.155	1329
6098	aminoglutethimide	17 µM	MCF7	-0.96	-0.143	0.161	7421
6099	alvespimycin	100 nM	HL60	-0.963	-0.157	0.148	1154
6100	digoxin	5 µM	MCF7	-1	-0.138	0.179	5324

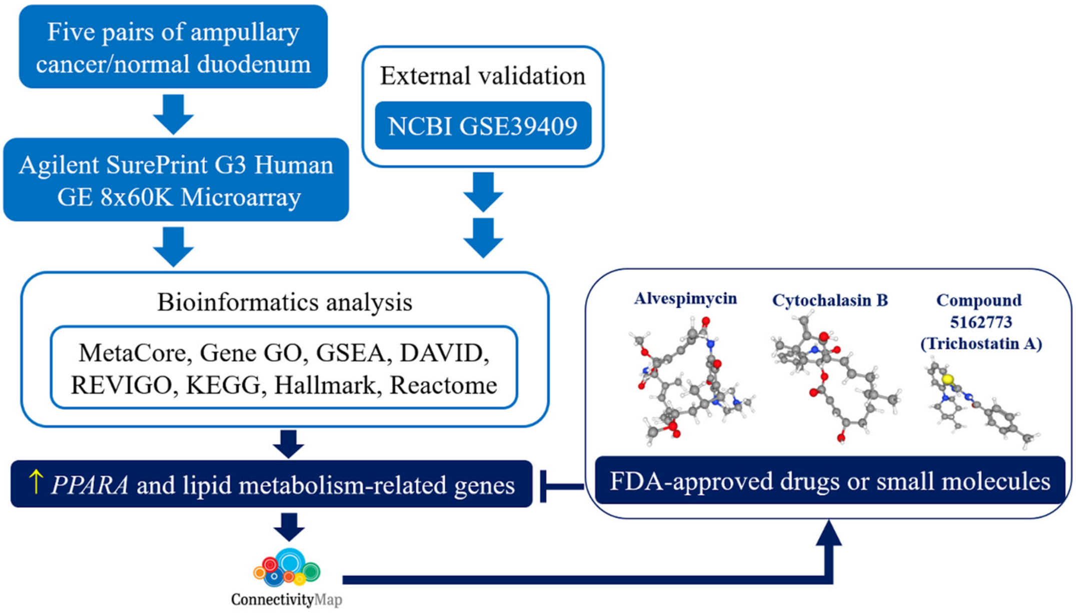

Supplementary Figure 1. Analytical algorithm used in the present study. The study began with a complementary (c)DNA microarray of patient samples validated by the National Center for Biotechnology Information (NCBI) dataset. Upregulated genes, peroxisome proliferator-activated receptor alpha (*PPARA*), and lipid metabolism-related genes were explored with the Connectivity Map (CMap) to find potential drugs.

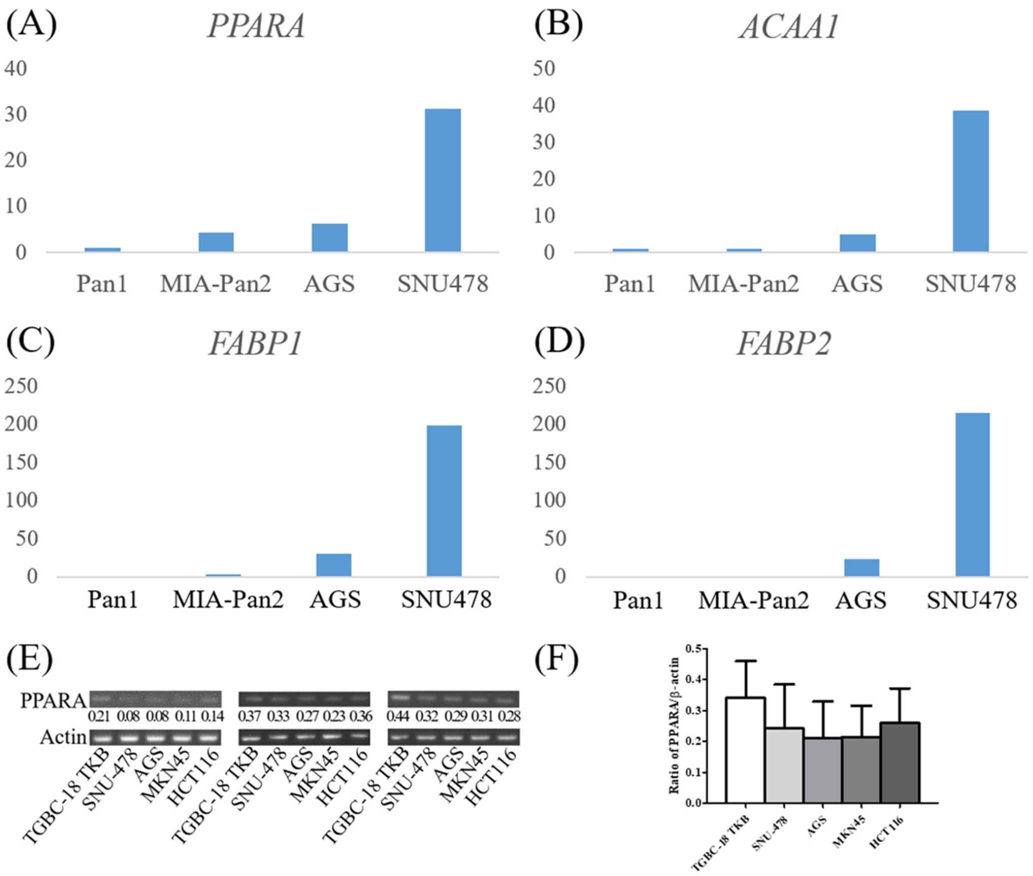

Supplementary Figure 2. Validation of target genes in cell lines from ampullary cancer and other cancers. mRNA expression levels of targeted genes from Figure 7 were examined in cell lines of different cancer types. (A) Expressions of *PPARA,* (B) *ACAA1*, (C) *FABP1*, and (D) *FABP2* by a qPCR. The graph represents a one-time experiment. (E) Expression of *PPARA* by a semiquantitative RT-PCR. β-actin served as a loading control. (F) Ratios of *PPARA*/β-actin in (E). The graph represents three independent experiments. Ampullary cancer cell lines: TGBC-18 TKB and SNU-478. Gastric cancer cell lines: AGS and MKN45. Pancreatic cancer cell lines: Pan1 and MIA-Pan2. Colon cancer cell line: HCT116.

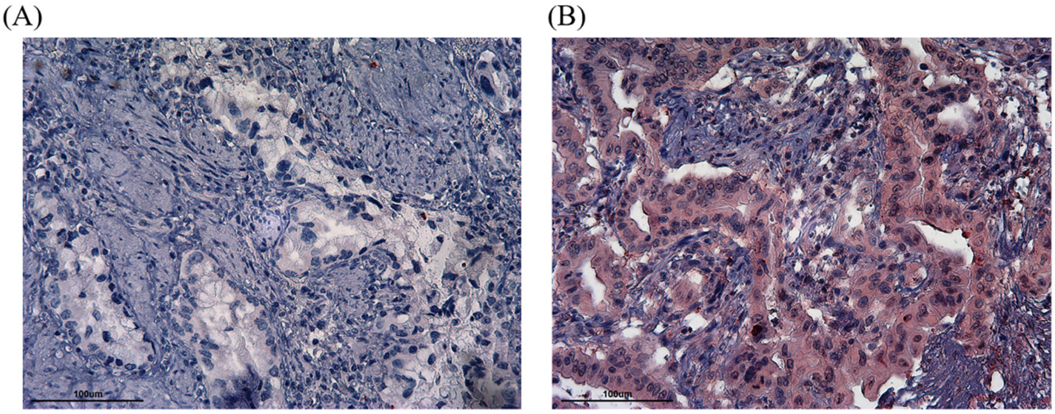

Supplementary Figure 3. Expression of peroxisome proliferator-activated receptor alpha (PPAR-α) protein in clinical samples of ampullary cancer by IHC staining. (A) Low expression of PPAR-α (×200). (B) High expression of PPAR-α (×200).

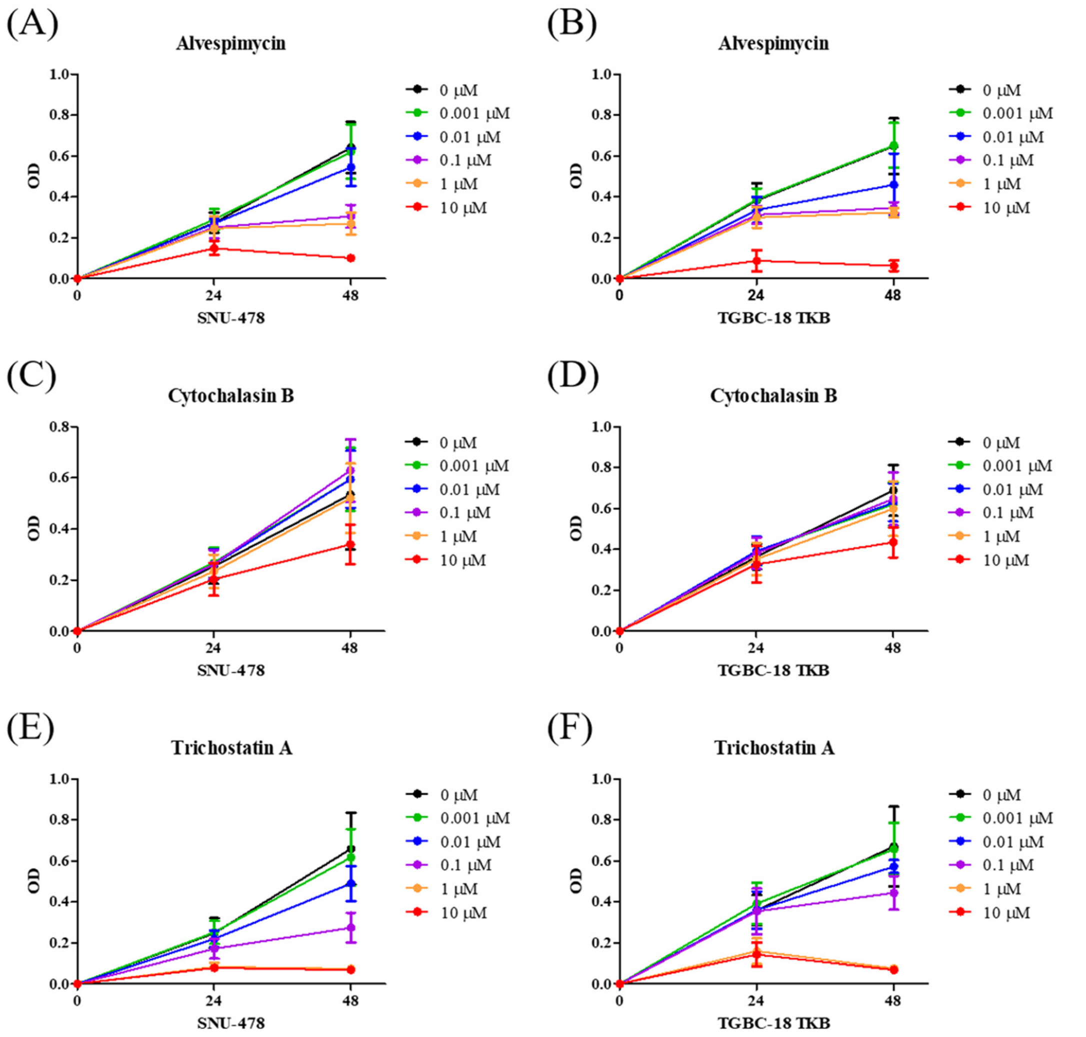

Supplementary Figure 4. Cell proliferation assay after treatment with potential drugs from Connectivity Map (CMap). An MTT assay was performed. (A) SNU-478 ampullary cancer cells treated with alvespimycin. (B) TGBC-18 TKB ampullary cancer cells treated with alvespimycin. (C) SNU-478 ampullary cancer cells treated with cytochalasin B. (D) TGBC-18 TKB ampullary cancer cells treated with cytochalasin B. (E) SNU-478 ampullary cancer cells treated with trichostatin A. (F) TGBC-18 TKB ampullary cancer cells treated with trichostatin A. Each graph represents three independent experiments.

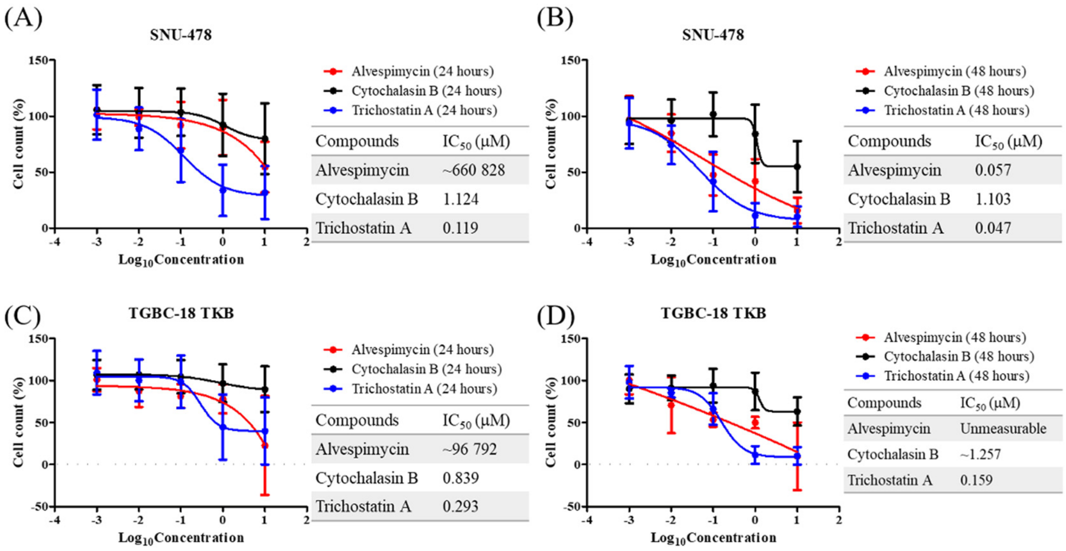

Supplementary Figure 5. Effects of potential drugs on ampullary cancer cells. Ampullary cancer cells were treated with alvespimycin, cytochalasin B, or trichostatin A for 24 or 48 h, and cell growth was evaluated by an MTT assay. (A) SNU-478 cells treated for 24 h. (B) SNU-478 cells treated for 48 h. (C) TGBC-18 TKB cells treated for 24 h. (D) TGBC-18 TKB cells treated for 48 h. Values of the dose-response growth inhibitory concentration (IC_{50}) were calculated according to a nonlinear regression curve with a variable slope. Graphs from three independent experiments with mean IC_{50} values ± standard deviation (SD) are shown.
